# Management of Cutaneous Dermatomyositis With Systemic Biologic Therapies: A Systematic Review

**DOI:** 10.1177/12034754241265717

**Published:** 2024-07-26

**Authors:** Siddhartha Sood, Edgar Akuffo-Addo, Abrahim Abduelmula, Martin Heung, David O. Croitoru, Vincent Piguet

**Affiliations:** 1 Temerty Faculty of Medicine, University of Toronto, Toronto, ON, Canada; 2 Division of Dermatology, Department of Medicine, University of Toronto, Toronto, ON, Canada; 3 Faculty of Health Sciences, McMaster University, Hamilton, ON, Canada; 4 Division of Dermatology, Department of Medicine, Women’s College Hospital, Toronto, ON, Canada

**Keywords:** dermatomyositis, biologics, treatment, therapeutics, systematic review, evidence-based

Dermatomyositis (DM) is a multisystem immune-mediated disease characterized by photosensitive erythema and poikiloderma with evidence of perifascicular myopathy.^1^ Treatment of DM remains challenging with no U.S. Food and Drug Administration approved targeted therapies. This systematic review investigates specific biologic agents for cutaneous and muscular manifestations of DM.

Following Preferred Reporting Items for Systematic reviews and Meta-Analyses criteria, MEDLINE and Embase databases were searched on March 15, 2024, using variations of specific keywords (Supplemental Table 1). Quality was assessed using the Oxford Centre for Evidence-Based Medicine Levels of Evidence 2011. After independent screening by 2 reviewers, 91 studies (publication date: 2003-2024) reflecting 569 patients and 589 treatments were included (Supplemental Figure 1 and Supplemental Table 2). The mean age was 34.6 (range: 4-76) years. Sex was reported in 342 patients [96 (28.1%) males and 246 females (71.9%)].

The most frequently used systemic biologics were: rituximab (68.3%, 402/589), infliximab (16%, 94/589), etanercept (5.6%, 33/589), and abatacept (3.6%, 21/589). Treatment duration was described in 452 cases (mean: 35 weeks; range: 1-156). Cutaneous disease was reported to be improved, no improvement, and worsened in 75.9% (447/589), 22.9% (135/589), and 1.2% (7/589) of cases, respectively (Supplemental Table 3). Concomitant systemic therapies were utilized in 29.9% (176/589) of cases, majority being systemic corticosteroids (14.1%, 83/589).

The studies used Cutaneous Dermatomyositis Area and Severity Index (CDASI), Skin Disease Activity Score (Skin-DAS), and Skin Visual Analog Scale (Skin-VAS) as outcome measures for skin involvement, demonstrating overall improvements of 24% (40/589), 59.7% (59/589), and 47.5% (129/589), respectively. Infliximab (Skin-DAS: 75%, 39/94), rituximab (Skin-VAS: 47%, 120/402; CDASI: 19.4%, 18/402; skin-DAS: 0%, 10/402), abatacept (CDASI: 4.6%, 10/21; Skin-VAS: 54.6%, 9/21), etanercept (CDASI: 26%, 11/33), and anifrolumab (CDASI: 77.8%, 1/2) reported these metrics (Supplemental Table 3).

Based on the American College of Rheumatology criteria, a meaningful change in Manual Muscle Testing (MMT) is defined as a 10% improvement from baseline.^2^ In studies that reported MMT, the mean increase from baseline was 18.3% (reported in 73/589 cases). Infliximab (99.3%, 5/94) and rituximab (17.7%, 38/402) resulted in the highest MMT improvement (Supplemental Table 3).

Treatment-emergent adverse events occurred in 122 cases (20.7%); most common being urinary tract infections (25.4%, 31/122) and other unspecified infections (23%, 28/122). One (0.2%) patient on anakinra passed away due to aspiration pneumonia; this was unclear if treatment-related.

Infiltrating activated helper T-cells (CD4^+^ CD-40L^+^) have been found to be upregulated in DM plaques, with TNF (tumour necrosis factor)-α, IFN (interferon)-1, and IL (interleukin)-1 highly expressed in muscular tissue, supporting the rationale for TNF-α/IL-1 inhibition and IFN-1 downstream blockade in DM.^3,4^ Likewise, we observed overall cutaneous improvement rates between 80% and 100% with adalimumab, anakinra, anifrolumab, dupilumab, ustekinumab, and rituximab with frequent Skin-DAS, Skin-VAS, and CDASI improvement in infliximab, abatacept, and etanercept, respectively. Similarly, infliximab and rituximab provided favourable results for myositis as assessed via MMT. To date, published guidelines have recommended rituximab for refractory cutaneous DM with limited guidance on other biologics.^5^

This study is limited by concomitant medication use, reporting bias, and omission of nonspecific agents (eg, immunoglobulins). Nonetheless, we highlight evidence supporting biologic use for DM. Additional larger-scale, placebo-controlled studies are warranted.

## Supplemental Material

sj-docx-1-cms-10.1177_12034754241265717 – Supplemental material for Management of Cutaneous Dermatomyositis With Systemic Biologic Therapies: A Systematic Review Supplemental material, sj-docx-1-cms-10.1177_12034754241265717 for Management of Cutaneous Dermatomyositis With Systemic Biologic Therapies: A Systematic Review by Siddhartha Sood, Edgar Akuffo-Addo, Abrahim Abduelmula, Martin Heung, David O. Croitoru and Vincent Piguet in Journal of Cutaneous Medicine and Surgery

sj-docx-2-cms-10.1177_12034754241265717 – Supplemental material for Management of Cutaneous Dermatomyositis With Systemic Biologic Therapies: A Systematic Review Supplemental material, sj-docx-2-cms-10.1177_12034754241265717 for Management of Cutaneous Dermatomyositis With Systemic Biologic Therapies: A Systematic Review by Siddhartha Sood, Edgar Akuffo-Addo, Abrahim Abduelmula, Martin Heung, David O. Croitoru and Vincent Piguet in Journal of Cutaneous Medicine and Surgery

sj-docx-3-cms-10.1177_12034754241265717 – Supplemental material for Management of Cutaneous Dermatomyositis With Systemic Biologic Therapies: A Systematic Review Supplemental material, sj-docx-3-cms-10.1177_12034754241265717 for Management of Cutaneous Dermatomyositis With Systemic Biologic Therapies: A Systematic Review by Siddhartha Sood, Edgar Akuffo-Addo, Abrahim Abduelmula, Martin Heung, David O. Croitoru and Vincent Piguet in Journal of Cutaneous Medicine and Surgery

sj-docx-4-cms-10.1177_12034754241265717 – Supplemental material for Management of Cutaneous Dermatomyositis With Systemic Biologic Therapies: A Systematic Review Supplemental material, sj-docx-4-cms-10.1177_12034754241265717 for Management of Cutaneous Dermatomyositis With Systemic Biologic Therapies: A Systematic Review by Siddhartha Sood, Edgar Akuffo-Addo, Abrahim Abduelmula, Martin Heung, David O. Croitoru and Vincent Piguet in Journal of Cutaneous Medicine and Surgery
