# Supplementary material for: Management of Cutaneous Dermatomyositis With Systemic Biologic Therapies: A Systematic Review
Source: J Cutan Med Surg. 2024 Jul 26;28(5):490–1. doi: 10.1177/12034754241265717 (PMC11528838; doi:10.1177/12034754241265717)
Supplement: sj-docx-1-cms-10.1177_12034754241265717 – Supplemental material for Management of Cutaneous Dermatomyositis With Systemic Biologic Therapies: A Systematic Review [file sj-docx-1-cms-10.1177_12034754241265717.docx]

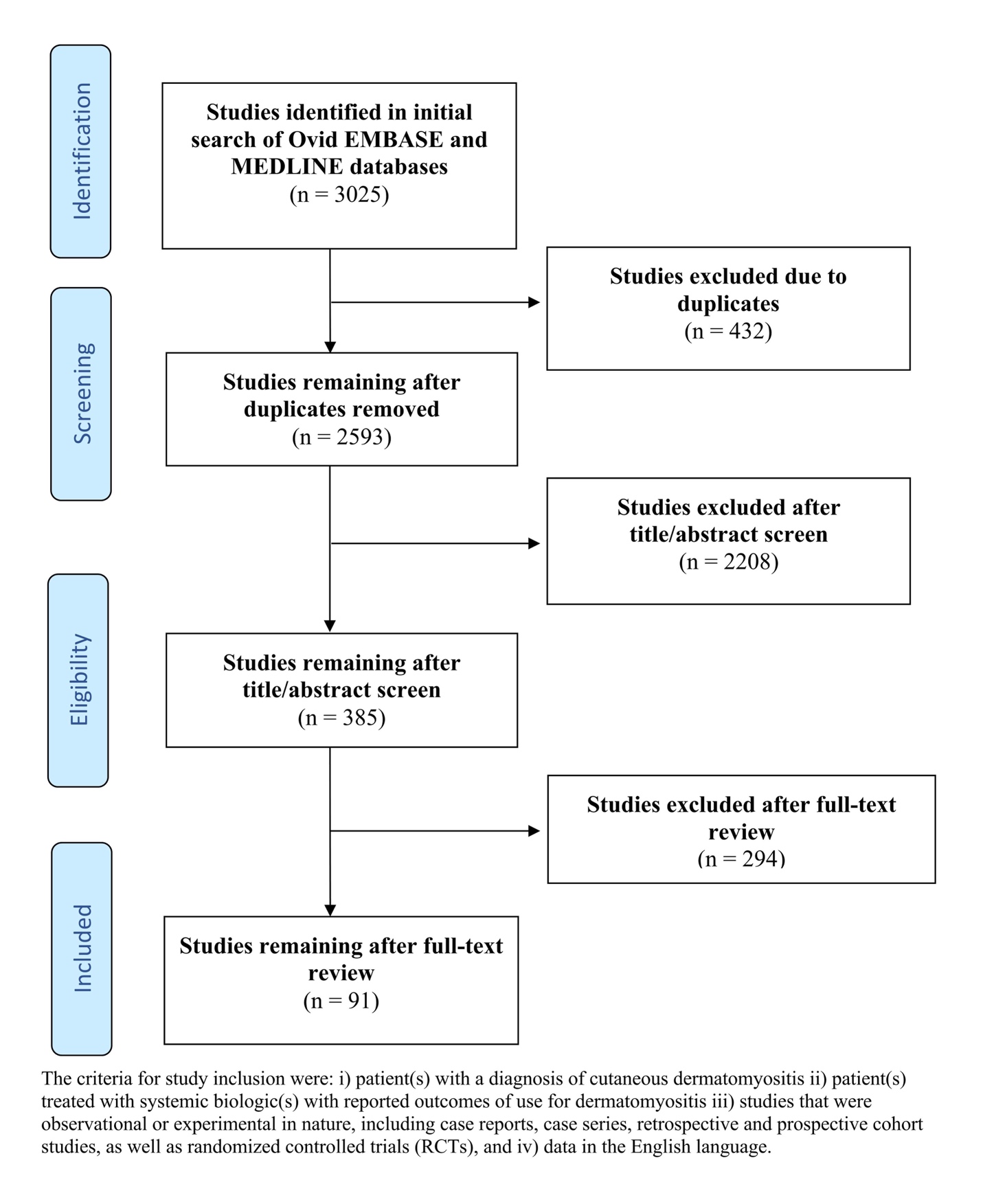


**Supplemental Figure 1.** Flow diagram of literature screening using the Preferred Reporting Items for Systematic Reviews and Meta-Analyses (PRISMA) guidelines. Figure adapted from http://prisma-statement.org.
