# Supplementary material for: Management of Cutaneous Dermatomyositis With Systemic Biologic Therapies: A Systematic Review
Source: J Cutan Med Surg. 2024 Jul 26;28(5):490–1. doi: 10.1177/12034754241265717 (PMC11528838; doi:10.1177/12034754241265717)
Supplement: sj-docx-2-cms-10.1177_12034754241265717 – Supplemental material for Management of Cutaneous Dermatomyositis With Systemic Biologic Therapies: A Systematic Review [file sj-docx-2-cms-10.1177_12034754241265717.docx]

**Supplemental Table I.** Search strategy used for literature screening.

Database(s):

Embase Classic+Embase 1947 to 2024 March 15, Ovid MEDLINE(R) ALL 1946 to 2024 March 15

Search strategy:

| # | Searches | Results |
| --- | --- | --- |
| 1 | dermatomyositis.mp. [mp=ti, ab, hw, tn, ot, dm, mf, dv, kf, fx, dq, bt, nm, ox, px, rx, ui, sy, ux, mx] | 37242 |
| 2 | biologic.mp. [mp=ti, ab, hw, tn, ot, dm, mf, dv, kf, fx, dq, bt, nm, ox, px, rx, ui, sy, ux, mx] | 201038 |
| 3 | rituximab.mp. [mp=ti, ab, hw, tn, ot, dm, mf, dv, kf, fx, dq, bt, nm, ox, px, rx, ui, sy, ux, mx] | 153598 |
| 4 | infliximab.mp. [mp=ti, ab, hw, tn, ot, dm, mf, dv, kf, fx, dq, bt, nm, ox, px, rx, ui, sy, ux, mx] | 84814 |
| 5 | etanercept.mp. [mp=ti, ab, hw, tn, ot, dm, mf, dv, kf, fx, dq, bt, nm, ox, px, rx, ui, sy, ux, mx] | 49730 |
| 6 | tocilizumab.mp. [mp=ti, ab, hw, tn, ot, dm, mf, dv, kf, fx, dq, bt, nm, ox, px, rx, ui, sy, ux, mx] | 36708 |
| 7 | sifalimumab.mp. [mp=ti, ab, hw, tn, ot, dm, mf, dv, kf, fx, dq, bt, nm, ox, px, rx, ui, sy, ux, mx] | 336 |
| 8 | belimumab.mp. [mp=ti, ab, hw, tn, ot, dm, mf, dv, kf, fx, dq, bt, nm, ox, px, rx, ui, sy, ux, mx] | 5610 |
| 9 | abatacept.mp. [mp=ti, ab, hw, tn, ot, dm, mf, dv, kf, fx, dq, bt, nm, ox, px, rx, ui, sy, ux, mx] | 18042 |
| 10 | ustekinumab.mp. [mp=ti, ab, hw, tn, ot, dm, mf, dv, kf, fx, dq, bt, nm, ox, px, rx, ui, sy, ux, mx] | 17533 |
| 11 | alemtuzumab.mp. [mp=ti, ab, hw, tn, ot, dm, mf, dv, kf, fx, dq, bt, nm, ox, px, rx, ui, sy, ux, mx] | 24313 |
| 12 | dupilumab.mp. [mp=ti, ab, hw, tn, ot, dm, mf, dv, kf, fx, dq, bt, nm, ox, px, rx, ui, sy, ux, mx] | 10488 |
| 13 | anakinra.mp. [mp=ti, ab, hw, tn, ot, dm, mf, dv, kf, fx, dq, bt, nm, ox, px, rx, ui, sy, ux, mx] | 13254 |
| 14 | eculizumab.mp. [mp=ti, ab, hw, tn, ot, dm, mf, dv, kf, fx, dq, bt, nm, ox, px, rx, ui, sy, ux, mx] | 12971 |
| 15 | basiliximab.mp. [mp=ti, ab, hw, tn, ot, dm, mf, dv, kf, fx, dq, bt, nm, ox, px, rx, ui, sy, ux, mx] | 16150 |
| 16 | 2 or 3 or 4 or 5 or 6 or 7 or 8 or 9 or 10 or 11 or 12 or 13 or 14 or 15 | 521273 |
| 17 | 1 and 16 | 3025 |
| 18 | remove duplicates from 17 | 2593 |
