# Supplementary material for: Management of Cutaneous Dermatomyositis With Systemic Biologic Therapies: A Systematic Review
Source: J Cutan Med Surg. 2024 Jul 26;28(5):490–1. doi: 10.1177/12034754241265717 (PMC11528838; doi:10.1177/12034754241265717)
Supplement: sj-docx-3-cms-10.1177_12034754241265717 – Supplemental material for Management of Cutaneous Dermatomyositis With Systemic Biologic Therapies: A Systematic Review [file sj-docx-3-cms-10.1177_12034754241265717.docx]

**Supplemental Table 2.** Cases of dermatomyositis treated with systemic biologic(s). Abbreviations: AE, adverse event; AZA; azathioprine; BID, twice daily; BIW, twice weekly; CAS, case series; CDASI, cutaneous dermatomyositis area and severity index; CoH, prospective cohort study; CRS, case report; CS, corticosteroids; CsA, cyclosporine A; CYC, cyclophosphamide; DAS, disease activity score; F, female; HCQ, hydroxychloroquine; Ig, immunoglobulin; IL, intralesional; ILD, interstitial lung disease; IV, intravenous; IVIG, intravenous immunoglobulin; M, male; METHYLPRED, methylprednisolone; MMF, mycophenolate mofetil; MMT, manual muscle testing; MTX, methotrexate; MYC, mycophenolate; N, no; NOR, no response; NR, none reported; PGA, physician global assessment; PO, oral; PRED, prednisone; Q2W, every 2 weeks; Q3W, every 3 weeks; Q4W, every 4 weeks; Q8W, every 8 weeks; RS, retrospective study; RTX, rituximab; SC, subcutaneous; TID, three times a day; TIW, three times a week; TOP, topical; UNK, unknown; VAS, visual analogue scale; Y, yes

| **Study** | **Study design (level of evidence)** | **Sample size** | **Mean age** | **Sex (n)** | **Comorbidities (n)** | **Juvenile or adult-onset DM (n)** | **Previous treatments for indication (n)** | **Biologic(s) (n)** | **Biologic: dose, frequency** | **Concurrent topical and systemic non-biologic therapies [route, dose, and frequency]** | **Biologic with reported disease metrics** | **CDASI: pre-/post-treatment** | **Mean change in CDASI, %** | **Skin-VAS: pre-/post-treatment** | **Mean change in Skin-VAS, %** | **Skin-DAS: pre-/post-treatment** | **Mean change in Skin-DAS (%)** | **MMT: pre-/post-treatment** | **Mean change in MMT (%)** | **Treatment duration, weeks** | **Treatment outcomes (as per general impression) [IM, NIM, WOR]** | **Follow up period, months** | **Adverse events (n)** | **Treatment deaths (n)** |
| --- | --- | --- | --- | --- | --- | --- | --- | --- | --- | --- | --- | --- | --- | --- | --- | --- | --- | --- | --- | --- | --- | --- | --- | --- |
| Aeschlimann 2018^1^ | CRS (5) | 1 | 13 | F | None | Juvenile | MTX; PRED | Rituximab | 375 mg/m^2, Q1W | IVIG [IV, NR, NR]; plasma exchange [IV, NR, NR]; PRED [PO, NR, NR] | Rituximab | NR / NR | NR | NR / NR | NR | NR / NR | NR | 38 / 79 | 51.9% | NR | NIM | NR | NR | NR |
| Aggarwal 2017^2^ | RCT (1b) | 120 | 36.1 | M(36); F(84) | NR | Juvenile (48); Adult (72) | NR | Rituximab | 575-750 mg/m^2, Q1W | NR | Rituximab | NR / NR |  | 3.22 / 1.72 | -47% | NR / NR | NR | NR / NR | NR | 44 | IM (120) | 11 | NR | NR |
| Albakri 2022^3^ | CRS (5) | 1 | 23 | F | COVID-19 | Adult | Ig; METHYLPRED; MTX; PRED | Rituximab | NR | PRED [PO, 40-5mg, QD to Q1W] | Rituximab | NR / NR | NR | NR / NR | NR | NR / NR | NR | 122 / 135 | 9.6% | NR | IM | NR | NR | NR |
| Amato 2011^4^ | RCT (1b) | 11 | 43.4 | M(5); F(6) | NR | Adult (11) | PRED | Etanercept | 50mg, Q1W | NR | Etanercept | 11.9 / 8.8 | -26% | NR / NR | NR | NR / NR | NR | 72 / 75.5 | 4.6% | 52 | IM (5); NIM (1): WOR (5) | NR | Elevated ANA (1) | NR |
| Ang 2024^5^ | CRS (5) | 1 | 43 | F | Breast carcinoma | Adult | CS [TOPICAL]; IVIG; HCQ; PRED; ruxolitinib; tacrolimus | Anifrolumab | 300mg, Q4W | NR |  | NR | NR | NR | NR | NR | NR | NR | NR | 9 | IM | 5 | NR | NR |
| Arabshahi 2011^6^ | CRS (5) | 1 | 14 | F | Steatohepatitis | Juvenile | CS [SYSTEMIC]; CYC; Ig; METHYLPRED; tacrolimus | 1. Infliximab / 2. Abatacept | 1. NR / 2. (NR, 10 mg/kg, Q2W to Q4W) | Sodium thiosulfate [IV, 10-15g, TIW to BIW]; sodium thiosulfate [TOP, 3%-10%, NR] | Abatacept | NR / NR | NR | NR / NR | NR | NR / NR | NR | 70 / 62 | -12.9% | NR / 24 | NIM / IM | 6 | NR / NR | NR / NR |
| Argobi 2021^7^ | CRS (5) | 1 | 37 | F | Cutaneous lupus; lichen planus | Adult (1) | HCQ; PRED; MTX; dapsone | Rituximab | 1000 mg IV, NR for 6 months | IVIG [IV, NR, NR]; MMF [UNK, NR, NR] | NR | NR / NR | NR | NR / NR | NR | NR / NR | NR | NR / NR | NR | NR | IM | NR | NR | NR |
| Bader-Meunier 2011^8^ | RS (2b) | 1 of 9 | 12 | F | GI Disease | Juvenile (1) | CsA; METHYLPRED; MMF; MTX; PRED | Rituximab | 375 mg/m^2, 2 doses | NR | NR | NR / NR | NR | NR / NR | NR | NR / NR | NR | NR / NR | NR | NR | IM | 30 | NR | NR |
| Bader-Meunier 2011^8^ | RS (2b) | 1 of 9 | 16 | F | GI Disease | Juvenile (1) | AZA; CsA; METHYLPRED; MMF; MTX; PRED; plasma exchange | Rituximab | 500 mg/m^2, 2 doses | NR | NR | NR / NR | NR | NR / NR | NR | NR / NR | NR | NR / NR | NR | NR | NIM | 26 | NR | NR |
| Bader-Meunier 2011^8^ | RS (2b) | 1 of 9 | 9 | M | GI Disease | Juvenile (1) | CsA; MTX; PRED; Ig | Rituximab | 375 mg/m^2, 4 doses | NR | NR | NR / NR | NR | NR / NR | NR | NR / NR | NR | NR / NR | NR | NR | NIM | 48 | NR | NR |
| Bader-Meunier 2011^8^ | RS (2b) | 1 of 9 | 8.1 | F | NR | Juvenile (1) | Ig; METHYLPRED; PRED; plasma exchange | Rituximab | 375 mg/m^2, 4 doses | NR | NR | NR / NR | NR | NR / NR | NR | NR / NR | NR | NR / NR | NR | NR | IM | 36 | NR | NR |
| Bader-Meunier 2011^8^ | RS (2b) | 1 of 9 | 7.1 | F | NR | Juvenile (1) | MTX; PRED | Rituximab |  | NR | NR | NR / NR | NR | NR / NR | NR | NR / NR | NR | NR / NR | NR | NR | IM | 42 | NR | NR |
| Bader-Meunier 2011^8^ | RS (2b) | 1 of 9 | 6.2 | F | NR | Juvenile (1) | CsA; MTX; PRED | Rituximab | 500 mg/m^2, 4 doses | NR | NR | NR / NR | NR | NR / NR | NR | NR / NR | NR | NR / NR | NR | NR | NIM | 24 | NR | NR |
| Bader-Meunier 2011^8^ | RS (2b) | 1 of 9 | 10.3 | F | NR | Juvenile (1) | CsA; METHYLPRED; MTX; PRED; plasma exchange | Rituximab | 375 mg/m^2, 4 doses | NR | NR | NR / NR | NR | NR / NR | NR | NR / NR | NR | NR / NR | NR | NR | NIM | 4 | NR | NR |
| Bader-Meunier 2011^8^ | RS (2b) | 1 of 9 | 11.5 | F | NR | Juvenile (1) | CsA; MTX; PRED | Rituximab | 375 mg/m^2, 4 doses | NR | NR | NR / NR | NR | NR / NR | NR | NR / NR | NR | NR / NR | NR | NR | NIM | 42 | NR | NR |
| Castano-Amores 2021^9^ | CRS (5) | 1 | 22 | F | Lupus | Adult (1) | HCQ; PRED | Rituximab | 1000 mg, Q2W | AZA [UNK, NR, NR] | NR | NR / NR | NR | NR / NR | NR | NR / NR | NR | NR / NR | NR | NR | IM | NR | Cerebral toxoplasmosis | NR |
| Chalhoub 2016^10^ | CRS (5) | 1 | 50 | F | NR | Adult (1) | AZA; CYC; etanercept; HCQ; Ig MTX; PRED; MMF | Infliximab | NR | NR | NR | NR / NR | NR | NR / NR | NR | NR / NR | NR | NR / NR | NR | NR | IM | 120 | Mycobacterial infection | NR |
| Chen 2013^11^ | CAS (4) | 1 of 14 | 52 | F | NR | Adult (1) | METHYLPRED | Infliximab | 5 mg/kg, Q2W to Q4W to Q8W | NR | NR | NR / NR | NR | NR / NR | NR | NR / NR | NR | NR / NR | NR | NR | IM | NR | NR | NR |
| Chen 2013^11^ | CAS (4) | 1 of 14 | 58 | F | NR | Adult (1) | METHYLPRED | Infliximab | 5 mg/kg, Q2W to Q4W to Q8W | NR | NR | NR / NR | NR | NR / NR | NR | NR / NR | NR | NR / NR | NR | NR | IM | NR | NR | NR |
| Chen 2013^11^ | CAS (4) | 1 of 14 | 55 | F | NR | Adult (1) | METHYLPRED | Infliximab | 5 mg/kg, Q2W to Q4W to Q8W | NR | NR | NR / NR | NR | NR / NR | NR | NR / NR | NR | NR / NR | NR | NR | IM | NR | NR | NR |
| Chen 2013^11^ | CAS (4) | 1 of 14 | 46 | F | NR | Adult (1) | METHYLPRED | Infliximab | 5 mg/kg, Q2W to Q4W to Q8W | NR | NR | NR / NR | NR | NR / NR | NR | NR / NR | NR | NR / NR | NR | NR | IM | NR | NR | NR |
| Chen 2013^11^ | CAS (4) | 1 of 14 | 42 | F | NR | Adult (1) | METHYLPRED | Infliximab | 5 mg/kg, Q2W to Q4W to Q8W | NR | NR | NR / NR | NR | NR / NR | NR | NR / NR | NR | NR / NR | NR | NR | IM | NR | NR | NR |
| Chen 2013^11^ | CAS (4) | 1 of 14 | 55 | F | NR | Adult (1) | METHYLPRED | Infliximab | 5 mg/kg, Q2W to Q4W to Q8W | NR | NR | NR / NR | NR | NR / NR | NR | NR / NR | NR | NR / NR | NR | NR | IM | NR | NR | NR |
| Chen 2013^11^ | CAS (4) | 1 of 14 | 43 | F | NR | Adult (1) | METHYLPRED | Infliximab | 5 mg/kg, Q2W to Q4W to Q8W | NR | NR | NR / NR | NR | NR / NR | NR | NR / NR | NR | NR / NR | NR | NR | IM | NR | NR | NR |
| Chen 2013^11^ | CAS (4) | 1 of 14 | 50 | F | NR | Adult (1) | METHYLPRED | Infliximab | 5 mg/kg, Q2W to Q4W to Q8W | NR | NR | NR / NR | NR | NR / NR | NR | NR / NR | NR | NR / NR | NR | NR | IM | NR | NR | NR |
| Chen 2013^11^ | CAS (4) | 1 of 14 | 52 | F | NR | Adult (1) | METHYLPRED | Infliximab | 5 mg/kg, Q2W to Q4W to Q8W | NR | NR | NR / NR | NR | NR / NR | NR | NR / NR | NR | NR / NR | NR | NR | IM | NR | NR | NR |
| Chen 2013^11^ | CAS (4) | 1 of 14 | 54 | F | NR | Adult (1) | METHYLPRED | Infliximab | 5 mg/kg, Q2W to Q4W to Q8W | NR | NR | NR / NR | NR | NR / NR | NR | NR / NR | NR | NR / NR | NR | NR | IM | NR | NR | NR |
| Chen 2013^11^ | CAS (4) | 1 of 14 | 49 | F | NR | Adult (1) | METHYLPRED | Infliximab | 5 mg/kg, Q2W to Q4W to Q8W | NR | NR | NR / NR | NR | NR / NR | NR | NR / NR | NR | NR / NR | NR | NR | NIM | NR | NR | NR |
| Chen 2013^10^ | CAS (4) | 1 of 14 | 63 | F | NR | Adult (1) | METHYLPRED | Infliximab | 5 mg/kg, Q2W to Q4W to Q8W | NR | NR | NR / NR | NR | NR / NR | NR | NR / NR | NR | NR / NR | NR | NR | NIM | NR | NR | NR |
| Chen 2013^11^ | CAS (4) | 1 of 14 | 63 | F | NR | Adult (1) | METHYLPRED | Infliximab | 5 mg/kg, Q2W to Q4W to Q8W | NR | NR | NR / NR | NR | NR / NR | NR | NR / NR | NR | NR / NR | NR | NR | NIM | NR | NR | NR |
| Chen 2013^11^ | CAS (4) | 1 of 14 | 52 | F | NR | Adult (1) | METHYLPRED | Infliximab | 5 mg/kg, Q2W to Q4W to Q8W | NR | NR | NR / NR | NR | NR / NR | NR | NR / NR | NR | NR / NR | NR | NR | NIM | NR | NR | NR |
| Choi 2009^12^ | CRS (5) | 1 | 46 | F | NR | Adult (1) | AZA; CYC; PRED; MTX | Etanercept | 25 mg, BIW | CS [PO, NR, NR]; MTX [UNK, NR, NR] | NR | NR / NR | NR | NR / NR | NR | NR / NR | NR | NR / NR | NR | NR | IM | NR | Necrotizing fasciitis | NR |
| Chung 2007^13^ | RCT (1b) | 1 of 8 | 42 | F | NR | Adult (1) | AZA; CsA; etanercept; MTX; tacrolimus | Rituximab | NR | PRED [PO, NR, NR]; MTX [UNK, NR, NR] | Rituximab | 6.6 / 10 | -51.5% | NR / NR | NR | NR / NR | NR | 68 / 72 | 5.6% | 24 | NIM | 12 | Infections requiring oral antibiotics | NR |
| Chung 2007^13^ | RCT (1b) | 1 of 8 | 76 | M | NR | Adult (1) | CS [SYSTEMIC]; topical immunomodulators | Rituximab | NR | PRED [PO, NR, NR] | Rituximab | 16.3 / 15.3 | -6.5% | NR / NR | NR | NR / NR | NR | 74.5 / 77 | 3.2% | 24 | NIM | 12 | Malignancy (colon and lung) | NR |
| Chung 2007^13^ | RCT (1b) | 1 of 8 | 42 | M | NR | Adult (1) | CS [SYSTEMIC]; MMF; topical immunomodulators | Rituximab | NR | MMF [UNK, NR, NR] | Rituximab | 7.4 / 5.5 | -25.7% | NR / NR | NR | NR / NR | NR | 77 / 79 | 2.5% | 24 | IM | 12 | Infections requiring oral antibiotics | NR |
| Chung 2007^13^ | RCT (1b) | 1 of 8 | 54 | M | NR | Adult (1) | CsA; HCQ; Ig; MTX | Rituximab | NR | METHYPRED [IV, NR, NR]; MTX [UNK, NR, NR] | Rituximab | 7 / 6 | -14.3% | NR / NR | NR | NR / NR | NR | 72 / 79 | 8.9% | 24 | IM | 12 | Infections requiring oral antibiotics | NR |
| Chung 2007^13^ | RCT (1b) | 1 of 8 | 65 | M | NR | Adult (1) | AZA; CS [SYSTEMIC]; HCQ | Rituximab | NR | AZA [PO, NR, NR]; HCQ [PO, NR, NR]; PRED [PO, NR, NR] | Rituximab | 5.1 / 3.5 | -31.4% | NR / NR | NR | NR / NR | NR | 77.5 / 79 | 1.9% | 24 | IM | 12 | Infections requiring oral antibiotics | NR |
| Chung 2007^13^ | RCT (1b) | 1 of 8 | 38 | M | NR | Adult (1) | AZA; HCQ; Ig; MTX | Rituximab | NR | NR | Rituximab | 2.5 / 2 | -20% | NR / NR | NR | NR / NR | NR | 74 / 76 | 2.6% | 24 | NIM | 12 | Infections requiring oral antibiotics | NR |
| Chung 2007^13^ | RCT (1b) | 1 of 8 | 42 | M | NR | Adult (1) | AZA; CsA; Ig; MMF; MTX; | Rituximab | NR | PRED [PO, NR, NR]; MTX [UNK, NR, NR] | Rituximab | 10.4 / 10.4 | 0 | NR / NR | NR | NR / NR | NR | 80 / 79 | -1.1% | 12 | NIM | 3 | Infections requiring oral antibiotics | NR |
| Chung 2007^13^ | RCT (1b) | 1 of 8 | 46 | M | NR | Adult (1) | AZA; MTX | Rituximab | NR | AZA [PO, NR, NR]; PRED [PO, NR, NR] | Rituximab | 9.2 / 6.5 | -29.3% | NR / NR | NR | NR / NR | NR | 72.5 / 65 | -10.3% | 24 | NIM | 12 | Infections requiring oral antibiotics | NR |
| Clottu 2012^14^ | CRS (5) | 1 | 68 | F | NR | Adult (1) | CYC; HCQ; Ig; MMF; PRED | Rituximab | 1,000 mg, Q2W for 2 weeks | CYC [IV, 600mg, 5 pulses] | NR | NR / NR | NR | NR / NR | NR | NR / NR | NR | NR / NR | NR | NR | IM | 3 | Papular necrosis | NR |
| Cooper 2007^15^ | RS (2b) | 1 of 4 | 10 | F | NR | Juvenile (1) | CYC; Ig; METHYLPRED; MTX; PRED | Rituximab | 375 mg/m^2, Q1W for 4 weeks | IVIG [IV, NR, NR]; MTX [UNK, NR, NR] | NR | NR / NR | NR | NR / NR | NR | NR / NR | NR | NR / NR | NR | NR | IM | 14 | NR | NR |
| Cooper 2007^15^ | RS (2b) | 1 of 4 | 14 | M | NR | Juvenile (1) | HCQ; MTX; PRED | Rituximab | 375 mg/m^2, Q1W for 4 weeks | PRED [PO, NR, NR]; METHYLPRED [IV, NR, NR]; MTX [UNK, NR, NR] | NR | NR / NR | NR | NR / NR | NR | NR / NR | NR | NR / NR | NR | NR | IM | 12 | NR | NR |
| Cooper 2007^15^ | RS (2b) | 1 of 4 | 14 | F | NR | Juvenile (1) | HCQ; Ig; METHYLPRED; MTX; PRED | Rituximab | 375 mg/m^2, Q1W for 4 weeks | IVIG [IV, NR, NR]; PRED [PO, NR, NR]; METHYLPRED [IV, NR, NR]; MTX [UNK, NR, NR] | NR | NR / NR | NR | NR / NR | NR | NR / NR | NR | NR / NR | NR | NR | IM | 12 | NR | NR |
| Cooper 2007^15^ | RS (2b) | 1 of 4 | 17 | F | NR | Juvenile (1) | MTX; PRED | Rituximab | 375 mg/m^2, Q1W for 4 weeks | METHYLPRED [IV, NR, NR]; MTX [UNK, NR, NR]; PRED [PO, NR, NR] | NR | NR / NR | NR | NR / NR | NR | NR / NR | NR | NR / NR | NR | NR | NIM | 10.5 | NR | NR |
| CrespoCruz 2021^16^ | CRS (5) | 1 | 49 | F | Thalassemia | Adult (1) | AZA; CS [SYSTEMIC]; CS [TOPICAL]; HCQ; Ig; mepacrine; MMF; MTX; oral tacrolimus | Rituximab | NR | HCQ [PO, NR, NR]; mepacrine [UNK, NR, NR]; MMF [UNK, NR, NR]; PRED [PO, NR, NR] | NR | NR / NR | NR | NR / NR | NR | NR / NR | NR | NR / NR | NR | NR | NIM | 7 | NR | NR |
| Curiel 2023^17^ | CoH (2b) | 10 | 12 | M(2); F(8) | NR | Juvenile (10) | PRED | Abatacept (10) | 125 mg, Q1W for 24 weeks | NR | Abatacept | 21.4 / 14 | –34.6% | NR / NR | NR | NR / NR | NR | NR / NR | NR | 24 | IM (9); NIM (1) | 6 | Worsening of pre-existing interstitial lung disease (requiring hospitalization and treatment) (1); worsening calcinosis (2); compression fracture (1); worsening right knee contracture (1); focal lipoatrophy (1); Febrile episodes (2); Skin infection (1); E. coli diarrhea (1) | NR |
| Day 2020^18^ | CRS (5) | 1 | 7 | F | NR | Juvenile (1) | NR | Rituximab | 600 mg, Q2W for a month | CYC [IV, 300-625 mg/m^2, Q2W]; IVIG [IV, 2g/kg, Q4W]; PRED [PO, 5mg, QD] | NR | NR / NR | NR | NR / NR | NR | NR / NR | NR | NR / NR | NR | NR | IM | NR | NR | NR |
| DeLorenzo 2021^19^ | CRS (5) | 1 | 55 | M | Acute myocardial infarction | Adult (1) | CS [SYSTEMIC]; Ig; METHYLPRED; MMF | Rituximab | NR | NR | NR | NR / NR | NR | NR / NR | NR | NR / NR | NR | NR / NR | NR | NR | NIM | 4 | Enterococal sepsis | NR |
| deSouza 2018^20^ | RS (2b) | 15 | 42.6 | NR | NR | Adult (38) | AZA; CsA; CYC; MMF MTX; leflunomide; and/or Ig | Rituximab | 1 g, Q2W for 4 weeks | Systemic immunosuppressants [UNK, NR, NR] | Rituximab | NR / NR | NR | NR / NR | NR | NR / NR | NR | 68.5 / 74 | 7.4% | 48 | IM (14); NIM (1) | 12 | NR | NR |
| Dinh 2007^21^ | CAS (4) | 1 of 3 | 22 | F | NR | Juvenile (1) | AZA; chloroquine; CsA; HCQ; leflunomide; MTX | Rituximab | 375 mg/m^2, Q1W for 4 weeks | betamethasone [TOP, NR, NR]; CS [IL, NR, NR]; PRED [PO, 5mg, QD] | NR | NR / NR | NR | NR / NR | NR | NR / NR | NR | NR / NR | NR | NR | IM | 24 | NR | NR |
| Dinh 2007^21^ | CAS (4) | 1 of 3 | 16 | F | NR | Juvenile (1) | CS [TOPICAL]; CsA; HCQ; MTX; PRED; topical tacrolimus | Rituximab | 375 mg/m^2, Q1W for 4 weeks | betamethasone dipropionate 0.05% [TOP, NR, NR]; CsA [PO, 100mg, BID] | NR | NR / NR | NR | NR / NR | NR | NR / NR | NR | NR / NR | NR | NR | IM | 20 | NR | NR |
| Dinh 2007^21^ | CAS (4) | 1 of 3 | 45 | F | NR | Adult (1) | AZA; HCQ; MMF; MTX; PRED | Rituximab | NR | pimecrolimus cream [TOP, NR, NR]; PRED [PO, NR, NR]; tacrolimus ointment [TOP, NR, NR] | NR | NR / NR | NR | NR / NR | NR | NR / NR | NR | NR / NR | NR | NR | IM | 6 | Seborrheic dermatitis | NR |
| Dold 2007^22^ | CAS (4) | 1 of 2 | 40 | F | NR | Adult (1) | Ig; METHYLPRED; MTX; plasmapheresis; PRED | Infliximab | 5 mg/kg, Q2W | HCQ [PO, 200 mg, BID]; MTX [UNK, 15mg, Q1W]; PRED [PO, 40mg, daily] | NR | NR / NR | NR | NR / NR | NR | NR / NR | NR | NR / NR | NR | NR | IM | 9 | NR | NR |
| Dold 2007^22^ | CAS (4) | 1 of 2 | 29 | F | NR | Adult (1) | PRED | Infliximab | 3 mg/kg, Q2W to Q4W | MTX [UNK, 15 mg, Q1W]; PRED [PO, 40mg, daily] | NR | NR / NR | NR | NR / NR | NR | NR / NR | NR | NR / NR | NR | NR | IM | 6 | NR | NR |
| Efthimiou 2006^23^ | RS (2b) | 1 of 8 | 33 | F | NR | Adult (1) | AZA; Ig; METHYLPRED; MTX; PRED | Etanercept | 25 mg, BIW | AZA [PO, NR, NR]; IVIG [IV, NR, NR]; METHYLPRED [IV, NR, NR]; MTX [UNK, NR, NR]; PRED [PO, NR, NR] | NR | NR / NR | NR | NR / NR | NR | NR / NR | NR | NR / NR | NR | NR | IM | 9 | NR | NR |
| Efthimiou 2006^23^ | RS (2b) | 1 of 8 | 44 | F | NR | Adult (1) | AZA; Ig; METHYLPRED; MTX PRED | Etanercept | 25 mg, BIW | IVIG [IV, NR, NR]; METHYLPRED [IV, NR, NR]; MTX [UNK, NR, NR]; PRED [PO, NR, NR] | NR | NR / NR | NR | NR / NR | NR | NR / NR | NR | NR / NR | NR | NR | IM | 8 | NR | NR |
| Efthimiou 2006^23^ | RS (2b) | 1 of 8 | 41 | F | NR | Adult (1) | AZA; Ig; METHYLPRED; MMF; MTX; PRED | Etanercept | 25 mg, BIW | AZA [PO, NR, NR]; IVIG [IV, NR, NR]; PRED [PO, NR, NR] | NR | NR / NR | NR | NR / NR | NR | NR / NR | NR | NR / NR | NR | NR | IM | 12 | Recurrent esophagitis | NR |
| Efthimiou 2006^23^ | RS (2b) | 1 of 8 | 68 | M | NR | Adult (1) | AZA; Ig; METHYLPRED; MTX; PRED | Etanercept | 25 mg, BIW | AZA [PO, NR, NR]; IVIG [IV, NR, NR]; METHYLPRED [IV, NR, NR]; MTX [UNK, NR, NR]; PRED [PO, NR, NR] | NR | NR / NR | NR | NR / NR | NR | NR / NR | NR | NR / NR | NR | NR | NIM | 5 | Nasal congestion; cardiac irregularity | NR |
| Efthimiou 2006^23^ | RS (2b) | 1 of 8 | 60 | M | NR | Adult (1) | AZA; Ig; METHYLPRED; PRED | Etanercept | 25 mg, BIW | IVIG [IV, NR, NR]; METHYLPRED [IV, NR, NR]; PRED [PO, NR, NR] | NR | NR / NR | NR | NR / NR | NR | NR / NR | NR | NR / NR | NR | NR | IM | 24 | NR | NR |
| Efthimiou 2006^23^ | RS (2b) | 1 of 8 | 70 | F | NR | Adult (1) | AZA; CYC; Ig; METHYLPRED; PRED | Etanercept | 25 mg, BIW | IVIG [IV, NR, NR]; METHYLPRED [IV, NR, NR]; PRED [PO, NR, NR] | NR | NR / NR | NR | NR / NR | NR | NR / NR | NR | NR / NR | NR | NR | IM | 20 | Splenic tumour/ascites | NR |
| Efthimiou 2006^23^ | RS (2b) | 1 of 8 | 41 | F | NR | Adult (1) | Ig; MTX; METHYLPRED; PRED | Infliximab | 3 mg/kg, Q2W | IVIG [IV, NR, NR]; MTX [UNK, NR, NR]; PRED [PO, NR, NR] | NR | NR / NR | NR | NR / NR | NR | NR / NR | NR | NR / NR | NR | NR | IM | 14 | NR | NR |
| Efthimiou 2006^23^ | RS (2b) | 1 of 8 | 73 | M | NR | Adult (1) | AZA; Ig; leflunomide METHYLPRED; MTX; PRED | Infliximab | 3 mg/kg, Q2W | IVIG [IV, NR, NR]; METHYLPRED [IV, NR, NR]; MTX [UNK, NR, NR]; PRED [PO, NR, NR] | NR | NR / NR | NR | NR / NR | NR | NR / NR | NR | NR / NR | NR | NR | NIM | NR | NR | NR |
| Fernandez 2009^24^ | CAS (4) | 1 of 4 | 41 | M | NR | Adult (1) | AZA; HCQ; Ig; METHYLPRED; PRED | Rituximab | 375 mg/m^2, Q1W for 4 weeks | METHYLPRED [UNK, 500mg, NR] | NR | NR / NR | NR | NR / NR | NR | NR / NR | NR | NR / NR | NR | NR | IM | 36 | NR | NR |
| Fernandez 2009^24^ | CAS (4) | 1 of 4 | 67 | M | NR | Adult (1) | HCQ; Ig; mepacrine; METHYLPRED | Rituximab | 375 mg/m^2, Q1W for 4 weeks | NR | NR | NR / NR | NR | NR / NR | NR | NR / NR | NR | NR / NR | NR | NR | IM | 36 | NR | NR |
| Fernandez 2009^24^ | CAS (4) | 1 of 4 | 42 | F | NR | Adult (1) | HCQ; Ig; mepacrine | Rituximab | 376 mg/m^2, Q1W for 4 weeks | NR |  | NR / NR | NR | NR / NR | NR | NR / NR | NR | NR / NR | NR | NR | IM | NR | NR | NR |
| Fernandez 2009^24^ | CAS (4) | 1 of 4 | 47 | F | NR | Adult (1) | AZA; CsA; CYC; etanercept; Ig; infliximab; mepacrine; MTX; PRED | Rituximab | 375 mg/m^2, Q1W for 4 weeks | METHYLPRED [UNK, 500mg, NR] |  | NR / NR | NR | NR / NR | NR | NR / NR | NR | NR / NR | NR | NR | IM | 36 | NR | NR |
| Fetter 2020^25^ | CRS (5) | 1 | 40 | F | NR | Adult (1) | AZA; Ig etanercept; MTX; PRED | Rituximab | 750 mg IV, 4 doses | NR | NR | NR / NR | NR | NR / NR | NR | NR / NR | NR | NR / NR | NR | NR | NIM | 3 | NR | NR |
| Ge 2021^26^ | CAS (4) | 1 of 11 | 36 | F | NR | Adult (1) | Calcineurin inhibitors; CYC; MMF; PRED | Rituximab | 375 mg/m^2, Q2W | Calcineurin inhibitors [UNK, NR, NR]; MMF [UNK, NR NR]; PRED [PO, NR, NR] | NR | NR / NR | NR | NR / NR | NR | NR / NR | NR | NR / NR | NR | NR | NIM | NR | NR | NR |
| Ge 2021^26^ | CAS (4) | 1 of 11 | 59 | M | NR | Adult (1) | PRED | Rituximab | 375 mg/m^2, Q2W | calcineurin inhibitors [UNK, NR, NR]; IVIG [IV, NR NR]; PRED [PO, NR, NR] | NR | NR / NR | NR | NR / NR | NR | NR / NR | NR | NR / NR | NR | NR | IM | NR | NR | NR |
| Ge 2021^26^ | CAS (4) | 1 of 11 | 24 | M | NR | Adult (1) | Calcineurin inhibitors; CYC; MTX; PRED | Rituximab | 375 mg/m^2, Q2W | Calcineurin inhibitors [UNK, NR, NR]; PRED [PO, NR, NR] | NR | NR / NR | NR | NR / NR | NR | NR / NR | NR | NR / NR | NR | NR | IM | NR | NR | NR |
| Ge 2021^26^ | CAS (4) | 1 of 11 | 41 | F | NR | Adult (1) | CYC; Ig; PRED | Rituximab | 375 mg/m^2, Q2W | CYC [UNK, NR, NR]; PRED [PO, NR, NR] | NR | NR / NR | NR | NR / NR | NR | NR / NR | NR | NR / NR | NR | NR | NIM | NR | NR | NR |
| Ge 2021^26^ | CAS (4) | 1 of 11 | 37 | F | NR | Adult (1) | CYC; Ig; MMF; PRED | Rituximab | 375 mg/m^2, Q2W | IVIG [IV, NR, NR]; PRED [PO, NR, NR] | NR | NR / NR | NR | NR / NR | NR | NR / NR | NR | NR / NR | NR | NR | NIM | NR | NR | NR |
| Ge 2021^26^ | CAS (4) | 1 of 11 | 51 | M | NR | Adult (1) | AZA; CYC; METHYLPRED | Rituximab | 375 mg/m^2, Q2W | IVIG [IV, NR, NR]; PRED [PO, NR, NR] | NR | NR / NR | NR | NR / NR | NR | NR / NR | NR | NR / NR | NR | NR | IM | NR | NR | NR |
| Ge 2021^26^ | CAS (4) | 1 of 11 | 56 | F | NR | Adult (1) | Calcineurin inhibitors; CYC; METHYLPRED | Rituximab | 375 mg/m^2, Q2W | Calcineurin inhibitors [UNK, NR, NR]; PRED [PO, NR, NR] | NR | NR / NR | NR | NR / NR | NR | NR / NR | NR | NR / NR | NR | NR | IM | NR | NR | NR |
| Ge 2021^26^ | CAS (4) | 1 of 11 | 53 | F | NR | Adult (1) | CYC; PRED | Rituximab | 100 mg, Q1W | Calcineurin inhibitors [UNK, NR, NR]; PRED [PO, NR, NR] | NR | NR / NR | NR | NR / NR | NR | NR / NR | NR | NR / NR | NR | NR | IM | NR | NR | NR |
| Ge 2021^26^ | CAS (4) | 1 of 11 | 28 | M | NR | Adult (1) | Ig; METHYLPRED | Rituximab | 100 mg, Q1W | Calcineurin inhibitors [UNK, NR, NR]; PRED [PO, NR, NR] | NR | NR / NR | NR | NR / NR | NR | NR / NR | NR | NR / NR | NR | NR | IM | NR | NR | NR |
| Ge 2021^26^ | CAS (4) | 1 of 11 | 36 | F | NR | Adult (1) | Calcineurin inhibitors; Ig; METHYLPRED | Rituximab | 100 mg, Q1W | Calcineurin inhibitors [UNK, NR, NR]; PRED [PO, NR, NR] | NR | NR / NR | NR | NR / NR | NR | NR / NR | NR | NR / NR | NR | NR | IM | NR | NR | NR |
| Ge 2021^26^ | CAS (4) | 1 of 11 | 39 | M | NR | Adult (1) | CYC; MMF; PRED; thalidomide | Rituximab | 100 mg, Q1W | PRED [PO, NR, NR] | NR | NR / NR | NR | NR / NR | NR | NR / NR | NR | NR / NR | NR | NR | IM | NR | NR | NR |
| Groh 2015^27^ | CRS (5) | 1 | 53 | F | NR | Adult | AZA; HCQ; IVIG; MTX; PRED | 1. Rituximab / 2. Anakinra | 1. (NR, NR) / 2. (100 mg, QD) | colchicine [PO, NR, NR]; IVIG [IV, 2 g/kg, Q4W]; MTX [PO, 15 mg, Q1W]; PRED [NR, 5 mg, QD] | NR | NR / NR | NR | NR / NR | NR | NR / NR | NR | NR / NR | NR | NR / NR | NIM / IM | 5 | NR / Aspiration pneumonia leading to death (authors uncertain if related to anakinra use) | NR / 1 of 1 |
| Grundtman 2008^28^ | CoH (2b) | 1 of 4 | 50 | M | NR | Adult (1) | CsA; MTX; PRED | Infliximab | 5 mg/kg, Q2W to Q4W to Q8W | MTX [UNK, 15mg, Q1W] | NR | NR / NR | NR | NR / NR | NR | NR / NR | NR | NR / NR | NR | NR | NIM | 4 | NR | NR |
| Grundtman 2008^28^ | CoH (2b) | 1 of 4 | 44 | M | NR | Adult (1) | AZA; CsA; Ig; MTX; PRED; | Infliximab | 5 mg/kg, Q2W to Q4W to Q8W | AZA [PO, 150mg, NR]; IVIG [IV, NR, NR]; PRED [PO, 5mg, NR] | NR | NR / NR | NR | NR / NR | NR | NR / NR | NR | NR / NR | NR | NR | NIM | 4 |  | NR |
| Grundtman 2008^28^ | CoH (2b) | 1 of 4 | 54 | F | NR | Adult (1) | AZA; PRED | Infliximab | 5 mg/kg, Q2W to Q4W to Q8W | AZA [PO, 150mg, NR]; MTX [UNK, 20mg, Q1W]; PRED [PO, 8mg, NR] | NR | NR / NR | NR | NR / NR | NR | NR / NR | NR | NR / NR | NR | NR | NIM | 4 |  | NR |
| Grundtman 2008^28^ | CoH (2b) | 1 of 4 | 74 | F | NR | Adult (1) | AZA; Ig MTX; PRED | Infliximab | 5 mg/kg, Q2W to Q4W to Q8W | AZA [PO, 150mg, NR]; PRED [PO, 10mg, NR] | NR | NR / NR | NR | NR / NR | NR | NR / NR | NR | NR / NR | NR | NR | NIM | 4 | NR | NR |
| Gullapalli 2020^29^ | CRS (5) | 1 | 12 | F | Pulmonary edema; renal injury; hemolytic anemia; thrombocytopenia purpura; Purtscher's retinopathy | Juvenile | Folic acid; Ig; METHYLPRED; MTX; plasmapheresis; PRED; vincristine | Rituximab | 375 mg/m^2, Q2W | METHYLPRED [IV, NR, NR]; IVIG [IV, NR, NR]; plasmapheresis [IV, NR, QD] | NR | NR / NR | NR | NR / NR | NR | NR / NR | NR | NR / NR | NR | 4 | IM | 72 | NR | NR |
| Haroon 2012^30^ | CRS (5) | 1 | 20 | F | NR | Adult (1) | CS [SYSTEMIC] | Rituximab | 1,000 mg, Q1W | NR | NR | NR / NR | NR | NR / NR | NR | NR / NR | NR | NR / NR | NR | NR | IM | 24 | NR | NR |
| Hassan 2021^31^ | CRS (5) | 1 | 54 | F | NR | Adult (1) | METHYLPRED; PRED | Infliximab | 5 mg/kg, Q2W to Q4W to Q8W | NR | NR | NR / NR | NR | NR / NR | NR | NR / NR | NR | NR / NR | NR | NR | IM | 3 | NR | NR |
| Hisanaga 2017^32^ | CRS (5) | 1 | 57 | F | NR | Adult (1) | CsA; CYC; PRED; | Rituximab | 550 mg, 4 doses | IVIG [IV, NR, NR] | NR | NR / NR | NR | NR / NR | NR | NR / NR | NR | NR / NR | NR | NR | IM | 3 | NR | NR |
| Holzer 2010^33^ | CAS (4) | 1 of 2 | 12 | F | NR | Juvenile (1) | CsA; Ig; METHYLPRED; MTX | Rituximab | 375 mg/m^2, Q1W for 4 weeks | MTX [UNK, NR, NR]; PRED [PO, 1mg/kg, QD] | NR | NR / NR | NR | NR / NR | NR | NR / NR | NR | NR / NR | NR | NR | NIM | NR | NR | NR |
| Holzer 2010^33^ | CAS (4) | 1 of 2 | 8 | F | NR | Juvenile (1) | Ig; METHYLPRED; MTX | Rituximab | 376 mg/m^2, Q1W for 3 weeks | CYC [IV, 750 mg/m^2, Q4W]; tacrolimus [PO, 0.2 mg/kg, QD] | NR | NR / NR | NR | NR / NR | NR | NR / NR | NR | NR / NR | NR | NR | NIM | NR | NR | NR |
| Huang 2019^34^ | CAS (4) | 1 of 8 | 52 | M | NR | NR | CS [SYSTEMIC]; CYC; mycophenolate | Rituximab | NR | NR | NR | NR / NR | NR | NR / NR | NR | NR / NR | NR | NR / NR | NR | NR | NIM | NR | NR | NR |
| Huang 2019^34^ | CAS (4) | 1 of 8 | 54 | F | NR | NR | CS [SYSTEMIC]; CYC; Ig | Rituximab | NR | NR | NR | NR / NR | NR | NR / NR | NR | NR / NR | NR | NR / NR | NR | NR | IM | NR | NR | NR |
| Huang 2019^34^ | CAS (4) | 1 of 8 | 59 | F | NR | NR | CS [SYSTEMIC]; CYC; mycophenolate | Rituximab | NR | NR | NR | NR / NR | NR | NR / NR | NR | NR / NR | NR | NR / NR | NR | NR | IM | NR | NR | NR |
| Huang 2019^34^ | CAS (4) | 1 of 8 | 43 | M | NR | NR | CS [SYSTEMIC]; CYC; rituximab | Rituximab | NR | NR | NR | NR / NR | NR | NR / NR | NR | NR / NR | NR | NR / NR | NR | NR | NIM | NR | NR | NR |
| Huang 2019^34^ | CAS (4) | 1 of 8 | 69 | M | NR | NR | CS [SYSTEMIC]; CYC | Rituximab | NR | NR | NR | NR / NR | NR | NR / NR | NR | NR / NR | NR | NR / NR | NR | NR | NIM | NR | NR | NR |
| Huang 2019^34^ | CAS (4) | 1 of 8 | 58 | F | NR | NR | Mycophenolate; CS [SYSTEMIC]; CsA; CYC; rituximab | Rituximab | NR | NR | NR | NR / NR | NR | NR / NR | NR | NR / NR | NR | NR / NR | NR | NR | NIM | NR | NR | NR |
| Huang 2019^34^ | CAS (4) | 1 of 8 | 46 | F | NR | NR | CS [SYSTEMIC]; CYC; Ig | Rituximab | NR | NR | NR | NR / NR | NR | NR / NR | NR | NR / NR | NR | NR / NR | NR | NR | NIM | NR | NR | NR |
| Huang 2019^34^ | CAS (4) | 1 of 8 | 44 | F | NR | NR | CS [SYSTEMIC]; mycophenolate | Rituximab | NR | NR | NR | NR / NR | NR | NR / NR | NR | NR / NR | NR | NR / NR | NR | NR | NIM | NR | NR | NR |
| Iannone 2006^35^ | CAS (4) | 1 of 5 | 39 | F | NR | NR | MTX; PRED | Etanercept | 25 mg, BIW | PRED [PO, NR, NR] [25mg, QD] | NR | NR / NR | NR | NR / NR | NR | NR / NR | NR | NR / NR | NR | 12 | NIM | 1 | NR | NR |
| Iannone 2006^35^ | CAS (4) | 1 of 5 | 59 | F | NR | NR | NR | Etanercept | 25 mg, BIW | PRED [PO, NR, NR] [25mg, QD] | NR | NR / NR | NR | NR / NR | NR | NR / NR | NR | NR / NR | NR | 12 | NIM | 3 | NR | NR |
| Iannone 2006^35^ | CAS (4) | 1 of 5 | 72 | M | NR | NR | AZA; PRED | Etanercept | 25 mg, BIW | PRED [PO, NR, NR] [30 mg, QD] | NR | NR / NR | NR | NR / NR | NR | NR / NR | NR | NR / NR | NR | 12 | NIM | 3 | NR | NR |
| Iannone 2006^35^ | CAS (4) | 1 of 5 | 63 | F | NR | NR | AZA; PRED | Etanercept | 25 mg, BIW | CsA [5 mg/kg, QD]; MTX [PO, 7.5mg, Q1W]; PRED [PO, NR, NR] [15 mg, QD] | NR | NR / NR | NR | NR / NR | NR | NR / NR | NR | NR / NR | NR | 12 | NIM | 3 | NR | NR |
| Iannone 2006^35^ | CAS (4) | 1 of 5 | 52 | F | NR | NR | CsA; PRED | Etanercept | 25 mg, BIW | PRED [PO, NR, NR] [10 mg, QD] | NR | NR / NR | NR | NR / NR | NR | NR / NR | NR | NR / NR | NR | 12 | NIM | 3 | NR | NR |
| Jin 2022^36^ | CRS (5) | 1 | 43 | F | Systemic contact dermatitis | Adult | Antihistamine; CS [TOPICAL] | Dupilumab | 600-300 mg, Q2W | NR | NR | NR / NR | NR | NR / NR | NR | NR / NR | NR | NR / NR | NR | 6 | IM | 1.5 | NR | NR |
| Jois 2011^37^ | CRS (5) | 1 | 35 | F | Tuberculosis | NR | AZA; Ig; MMF; MTX; PRED | Rituximab | 1 g, Q2W for 4 months | NR | NR | NR / NR | NR | NR / NR | NR | NR / NR | NR | NR / NR | NR | 16 | IM | 24 | NR | NR |
| Joshi 2011^38^ | CRS (5) | 1 | 46 | M | NR | NR | CYC; Ig; MMF; MTX; PRED | Rituximab | 1 g | METHYLPRED [UNK, 250 mg, Q2W] | NR | NR / NR | NR | NR / NR | NR | NR / NR | NR | NR / NR | NR | NR | IM | NR | NR | NR |
| Kaposztas 2008^39^ | CRS (5) | 1 | 65 | F | ruptured arteriovenous malformation; hypertension; atopic dermatitis; asthma | NR | AZA; MMF; PRED; sirolimus | Rituximab | 375 mg, Q1W for 4 weeks | Sirolimus [PO, 2mg, QD] | NR | NR / NR | NR | NR / NR | NR | NR / NR | NR | NR / NR | NR | NR | IM | 2 | NR | NR |
| Komiya 2017^40^ | CRS (5) | 1 | 57 | F | idiopathic thrombocytopenic purpura; acute heart failure | NR | Dexamethasone; etoposide; plasmapheresis PRED | Infliximab | 5 mg/kg, Q2W to Q4W to Q8W | PRED [PO, 5 mg, QD]; tacrolimus [PO, 3 mg, QD] | NR | NR / NR | NR | NR / NR | NR | NR / NR | NR | NR / NR | NR | NR | IM | NR | NR | NR |
| Kondo 2014^41^ | CRS (5) | 1 | 32 | F | NR | NR | Adalimumab; CYC; Ig; MTX; tacrolimus | Tocilizumab | NR | CS [PO, NR, NR] |  | NR / NR | NR | NR / NR | NR | NR / NR | NR | NR / NR | NR | NR | IM | NR | NR | NR |
| Kuye 2017^42^ | RS (2b) | 25 | NR | NR | NR | NR | NR | Rituximab | NR | NR | NR | NR / NR | NR | NR / NR | NR | NR / NR | NR | NR / NR | NR | NR | IM (18); NIM (7) | 6 | NR | NR |
| Lam 2018^43^ | CRS (5) | 1 | 40 | F | subclinical hypothyroidism | NR | CsA; PRED; tacrolimus | Rituximab | 375 mg/m^2, Q1W for 4 weeks | NR | NR | NR / NR | NR | NR / NR | NR | NR / NR | NR | NR / NR | NR | NR | NIM | NR | NR | NR |
| Levine 2005^44^ | CoH (2b) | 1 of 5 | 21 | F | NR | NR | AZA; CS [SYSTEMIC] | Rituximab | 375 mg/m^2, Q1W | IVIG [IV, NR, NR] | NR | NR / NR | NR | NR / NR | NR | NR / NR | NR | NR / NR | NR | NR | IM | 52 | NR | NR |
| Levine 2005^44^ | CoH (2b) | 1 of 5 | 53 | F | NR | NR | CS [SYSTEMIC]; CsA; etanercept; MTX | Rituximab | 375 mg/m^2, Q1W | AZA [PO, NR, NR]; IVIG [IV, NR, NR] | NR | NR / NR | NR | NR / NR | NR | NR / NR | NR | NR / NR | NR | NR | IM | 52 | NR | NR |
| Levine 2005^44^ | CoH (2b) | 1 of 5 | 38 | F | NR | NR | HCQ; Ig; MTX | Rituximab | 375 mg/m^2, Q1W | CS [SYSTEMIC] [UNK, NR, NR] | NR | NR / NR | NR | NR / NR | NR | NR / NR | NR | NR / NR | NR | NR | IM | 52 | NR | NR |
| Levine 2005^44^ | CoH (2b) | 1 of 5 | 53 | F | NR | NR | Etanercept; MTX | Rituximab | 375 mg/m^2, Q1W | AZA [PO, NR, NR]; CS [SYSTEMIC] [UNK, NR, NR] | NR | NR / NR | NR | NR / NR | NR | NR / NR | NR | NR / NR | NR | NR | IM | 52 | NR | NR |
| Levine 2005^44^ | CoH (2b) | 1 of 5 | 45 | M | NR | NR | AZA; CS [SYSTEMIC]; etanercept; MTX | Rituximab | 375 mg/m^2, Q1W | NR | NR | NR / NR |  | NR / NR | NR | NR / NR | NR | NR / NR | NR | NR | IM | 52 | NR | NR |
| Li 2019^45^ | CRS (5) | 1 | 63 | M | Diffuse large B-cell lymphoma | NR | CYC; METHYLPRED; PRED | Rituximab | NR | pirfenidone [PO, 0.6g, TID]; PRED [PO, 7.5mg, QD] | NR | NR / NR | NR | NR / NR | NR | NR / NR | NR | NR / NR | NR | NR | IM | NR | NR | NR |
| Lim 2021^46^ | CRS (5) | 1 | 58 | F | NR | NR | AZA; Ig; mycophenolate; MTX; PRED | Rituximab | 1g, Q1W for 2 weeks | METHYLPRED [UNK, 40 mg, QD] | NR | NR / NR | NR | NR / NR | NR | NR / NR | NR | NR / NR | NR | NR | NIM | NR | Altered mental status | NR |
| Lu 2021^47^ | CRS (5) | 1 | 44 | F | NR | NR | CYC | Tocilizumab | 480 mg, Q4W | METHYLPRED [PO, 40 mg, QD] | NR | NR / NR | NR | NR / NR | NR | NR / NR | NR | NR / NR | NR | 32 | IM | 12 | NR | NR |
| Lyakhovitsky 2020^48^ | CRS (5) | 1 | 68 | F | Hypertension; hypothyroidism; rheumatoid arthritis | NR | HCQ; MTX; tofacitinib | Etanercept | 50 mg, Q1W | NR | NR | NR / NR | NR | NR / NR | NR | NR / NR | NR | NR / NR | NR | 3 | NIM | 6 | NR | NR |
| Majmudar 2009^49^ | CAS (4) | 1 | 21 | F | NR | NR | AZA; Ig | Rituximab | 1000 mg, BIW for 1 month | METHYLPRED [IV, 100 mg, NR]; MTX [UNK, NR, NR] | NR | NR / NR | NR | NR / NR | NR | NR / NR | NR | NR / NR | NR | 26 | IM | 6 | NR | NR |
| Montoya 2017^50^ | CRS (5) | 1 | 20 | M | NR | NR | METHYLPRED; rituximab | Ustekinumab | 45 mg, Q4W to Q12W | NR | NR | NR / NR | NR | NR / NR | NR | NR / NR | NR | NR / NR | NR | 72 | IM | 18 | NR | NR |
| Munoz-Beamud 2013^51^ | CoH (2b) | 6 | NR | M(3); F(3) | NR | NR | AZA; Ig; MTX | Rituximab | 1g, Q2W | MTX [UNK, NR, NR]; PRED [PO, NR, NR] | NR | NR / NR | NR | NR / NR | NR | NR / NR | NR | NR / NR | NR | 52 | IM | 12 | NR | NR |
| Navarro-Navarro 2021^52^ | CAS (4) | 1 of 2 | 46 | F | NR | NR | Ig; METHYLPRED; MTX; tacrolimus | Rituximab | NR | NR | NR | NR / NR | NR | NR / NR | NR | NR / NR | NR | NR / NR | NR | NR | NIM | 5 | NR | NR |
| Navarro-Navarro 2021^52^ | CAS (4) | 1 of 2 | 53 | F | NR | NR | AZA; CsA; HCQ; Ig; METHYLPRED; MTX; MMF | Rituximab | NR | NR | NR | NR / NR | NR | NR / NR | NR | NR / NR | NR | NR / NR | NR | NR | NIM | 4 | NR | NR |
| Neri 2022^53^ | CRS (5) | 1 | 32 | F | Bilateral panuveitis | Adult | CS [SYSTEMIC]; METHYLPRED; MMF; PRED | Infliximab | 5 mg/kg, Q2W to Q4W to Q8W | MMF [PO, 1000 mg, BIW] | NR | NR / NR | NR | NR / NR | NR | NR / NR | NR | NR / NR | NR | 4 | IM | 11 | NR | NR |
| Noss 2006^54^ | CAS (4) | 1 of 3 | 54 | F | acute cholecystitis, congestive heart failure, and tachycardia | NR | AZA; Ig; MTX; PRED | Rituximab | 1000 mg, BIW for 1 month | MMF [PO, 500 mg, BID] | NR | NR / NR | NR | NR / NR | NR | NR / NR | NR | NR / NR | NR | 52 | IM | 12 | NR | NR |
| Noss 2006^54^ | CAS (4) | 1 of 3 | 54 | F | NR | NR | AZA; Ig; infliximab; MTX; PRED | Rituximab | 1000 mg, BIW for 1 month | METHYLPRED [IV, 100mg, BIW] | NR | NR / NR | NR | NR / NR | NR | NR / NR | NR | NR / NR | NR | 8 | IM | 2 | NR | NR |
| Noss 2006^54^ | CAS (4) | 1 of 3 | 48 | M | NR | NR | Ig; MTX; PRED | Rituximab | 1000 mg, BIW for 1 month | METHYLPRED [IV, 100mg, BIW] | NR | NR / NR | NR | NR / NR | NR | NR / NR | NR | NR / NR | NR | 26 | IM | 6 | NR | NR |
| O'Brien 2022^55^ | CRS (5) | 1 | 28 | F | NR | Adult | AZA; CS [SYSTEMIC]; CS [TOPICAL]; dapsone; HCQ; MTX; thalidomide | Dupilumab | 600-300 mg, Q2W | NR | NR | NR / NR | NR | NR / NR | NR | NR / NR | NR | NR / NR | NR | 20 | IM | 5 | NR | NR |
| Oddis 2013^56^ | RCT (1b) | 124 | 41.5 | NR | NR | NR | PRED; systemic immunosuppressive agent | Rituximab | 575-750 mg/m^2, Q1W | NR | NR | NR / NR | NR | NR / NR | NR | NR / NR | NR | NR / NR | NR | 44 | IM (99): NIM (25) | 11 | UTI (30); joint pain/swelling (3); hypersensitivity reactions (3) | NR |
| Oddis 2022^57^ | RCT (1b) | 10 | NR | NR | NR | Adult (10) | CS [SYSTEMIC] (10) | Tocilizumab | 8 mg/kg, Q4W] (10) | NR | Tocilizumab | NR / NR | NR | NR / NR | NR | NR / NR | NR | 126.7 / 135.1 | 6.2% | 24 | NIM (10) | 6 | Infection (8); bilateral pulmonary emboli (1) | NR |
| Ogawa 2017^58^ | CRS (5) | 1 | 48 | M | NR | NR | NR | Rituximab | 700 mg, Q1W for 4 weeks | CsA [PO, NR, NR]; METHYLPRED [IV, 1g, QD for 3 days]; PRED [PO, 60 mg, QD] | NR | NR / NR | NR | NR / NR | NR | NR / NR | NR | NR / NR | NR | 4 | IM | 4 | NR | NR |
| Paik 2017^59^ | CRS (5) | 1 | 55 | F | NR | NR | AZA; Ig MTX; mycophenolate | Rituximab | 1g, Q2W | PRED [PO, 10 mg, QD] | NR | NR / NR | NR | NR / NR | NR | NR / NR | NR | NR / NR | NR | NR | NIM | 6 | NR | NR |
| Pandya 2023^60^ | CAS (4) | 1 | 42 | F | Systemic lupus erythematosus | NR | AZA; CS [TOP]; CYC; dapsone; HCQ; METHYLPRED; MMF; quinacrine | Anakinra | 100mg, NR | CYC [NR, Q2W]; HCQ [PO, 400mg, QD]; METHYLPRED [UNK, 32mg, QD]; PRED [PO, 5mg, QD] |  | NR | NR | NR | NR | NR | NR | NR | NR | NR | IM | NR | NR | NR |
| Park 2012^61^ | CRS (5) | 1 | 48 | F | NR | NR | CsA; MTX; PRED | Adalimumab | 40 mg, Q2W | PRED [PO, 10mg, QD] | NR | NR / NR | NR | NR / NR | NR | NR / NR | NR | NR / NR | NR | 12 | IM | 3 | NR | NR |
| Rider 2014^62^ | RCT (1b) | 10 | 25.1 | M(3); F(7) | NR | NR | AZA; CsA; HCQ; MTX; mycophenolate | Rituximab | NR, Q1W | NR | Rituximab | 17.5 / 9.97 | -17 | NR / NR | NR | 6 / 6 | 0 | 54.5 / 69.76 | 21.9% | 44 | IM (4); NIM (6) | 11 | NR | NR |
| Riley 2008^63^ | CAS (4) | 4 | 4 | M(2); F(2) | NR | NR | NR | Infliximab | 3 mg/kg, Q2W to Q4W to Q8W | NR | NR | NR / NR | NR | NR / NR | NR | NR / NR | NR | NR / NR | NR | 104 | IM (4) | 24 | Infection (2) | NR |
| Rodziewicz 2018^64^ | CRS (5) | 1 | 70 | F | NR | NR | Ig; PRED | Rituximab | 1g, Q2W | CsA [PO, NR, NR]; IVIG [IV, NR, NR] | NR | NR / NR | NR | NR / NR | NR | NR / NR | NR | NR / NR | NR | 156 | IM | 36 | NR | NR |
| Rouster-Stevens 2014^65^ | CoH (2b) | 9 | 14 | M(2); F(7) | NR | NR | CsA; HCQ; METHYLPRED; MTX; PRED | Etanercept | 0.4 mg/kg, BIW for 12 weeks | HCQ [PO, NR, NR]; MTX [UNK, NR, NR] | NR | NR / NR | NR | NR / NR | NR | NR / NR | NR | NR / NR | NR | 12 | IM (7); NIM (1); WOR (1) | 6 | NR | NR |
| Sabbagh 2019^66^ | CAS (4) | 1 of 2 | 12 | M | NR | NR | Ig; METHYLPRED; MTX | Rituximab | NR | NR | Rituximab | NR / NR | NR | NR / NR | NR | NR / NR | NR | 60 / 78 | 23.1% | NR | NIM | 15 | NR | NR |
| Sabbagh 2019^66^ | CAS (4) | 1 of 2 | 15 | F | NR | NR | CYC; Ig; METHYLPRED | Rituximab | NR | NR | Rituximab | NR / NR | NR | NR / NR | NR | NR / NR | NR | 72 / 79 | 8.9% | NR | NIM | 12 | NR | NR |
| Sallum 2011^67^ | CRS (5) | 1 | 7 | M | NR | NR | CsA; HCQ; Ig; METHYLPRED; MTX; PRED; thalidomide | Rituximab | 375 mg/m^2, NR | Alendronate [PO, 70mg, Q1W]; CsA [PO, 5mg/kg, QD]; HCQ [PO, NR, NR]; IVIG [IV, NR, NR]; MTX [UNK,1mg/kg, Q1W]; sulphate [PO, 6.2mg/kg, QD]; thalidomide [PO, 2.3mg/kg, QD] | NR | NR / NR | NR | NR / NR | NR | NR / NR | NR | NR / NR | NR | 4 | IM | 6 | NR | NR |
| Sanchez-Ramon 2010^68^ | CRS (5) | 1 | 44 | F | NR | NR | AZA; METHYLPRED; MTX; PRED | Rituximab | 1g, Q2W | IVIG [IV, 2g/kg, Q1W] | NR | NR / NR | NR | NR / NR | NR | NR / NR | NR | NR / NR | NR | 8 | IM | 30 | NR | NR |
| Schiffenbauer 2018^69^ | RCT (1b) | 1 | 41.8 | NR | NR | NR | NR | Infliximab | 5 mg/kg, Q2W to Q4W to Q8W | NR | Infliximab | NR / NR | NR | NR / NR | NR | NR / NR | NR | 22 / 50 | 56% | 14 | IM | 3 | NR | NR |
| Schildt 2021^70^ | CAS (4) | 1 of 2 | 4 | F | NR | NR | Colchicine | Rituximab | 600mg, Q3W | IVIG [IV, 2g/kg, NR]; MTX [UNK, 15mg, Q1W]; PRED [PO, 30mg, QD] | NR | NR / NR | NR | NR / NR | NR | NR / NR | NR | NR / NR | NR | 3 | IM | 3 | NR | NR |
| Schildt 2021^70^ | CAS (4) | 1 of 2 | 11 | F | polyarticular JIA | NR | CS [SYSTEMIC] | Rituximab | 750 mg/m^2, NR | IVIG [IV, 2g/kg, NR]; METHYLPRED [PO, 30mg/kg, QD] | Rituximab | NR / NR | NR | NR / NR | NR | NR / NR | NR | 40 / 80 | 100% | 3 | IM | 3 | NR | NR |
| Shaw 2024^71^ | CRS (5) | 1 | 14 | F | NR | Juvenile | CS [SYSTEMIC]; MMF; HCQ; IVIG; MTX; CsA; tacrolimus; tofacitinib | 1. Rituximab / 2. Anifrolumab | 1. NR / 2. 300mg, Q4W | NR |  | 36 / 8 | -77.80% | NR | NR | NR | NR | NR | NR | 8 | IM | 2 | NR | NR |
| Simou 2020^72^ | RS (2b) | 15 of 54 | 5.2 | NR | NR | NR | AZA; HCQ; MTX | Adalimumab | 1. (6 mg/kg, Q4W) / 2. (24 mg/m^2, Q2W) | PRED [PO, 6mg, QD] | NR | NR / NR | NR | NR / NR | NR | NR / NR | NR | NR / NR | NR | 12 | NIM (15) / IM (15) | 12 | NR / NR | NR |
| Simou 2020^72^ | RS (2b) | 39 of 54 | 5.2 | NR | NR | NR | AZA; HCQ; MTX | Infliximab | 6 mg/kg, Q4W | PRED [PO, 6mg, QD] | Infliximab | NR / NR | NR | NR / NR | NR | 4 / 1 | -75% | NR / NR | NR | 12 | IM (39) | 12 | Allergic reactions to infliximab (9) | NR |
| Sozeri 2020^73^ | CRS (5) | 1 of 2 | 7 | M | 1440IU/I | NR | HCQ; MTX; PRED | Infliximab | NR | IVIG [IV, NR, NR]; MMF [UNK, NR, NR]; pamidronate [PO, NR, NR] | NR | NR / NR | NR | NR / NR | NR | NR / NR | NR | NR / NR | NR | 12 | NIM | 3 | NR | NR |
| Sozeri 2020^73^ | CRS (5) | 1 of 2 | 9 | F | NR | NR | HCQ; MTX; PRED | 1. Infliximab / 2. Rituximab | 1. (5mg, BID) / 2. (NR) | IVIG [IV, NR, NR]; MMF [UNK, NR, NR]; pamidronate [PO, NR, NR] | NR | NR / NR | NR | NR / NR | NR | NR / NR | NR | NR / NR | NR | NR / 12 | NIM / NIM | 3 | NR / NR | NR / NR |
| Speth 2016^74^ | CRS (5) | 1 | 10 | F | NR | Juvenile | CYC; Ig; MMF; MTX; PRED | Rituximab | 375mg/m^2, NR | Ig [SC, 70 g, Q4W] | NR | NR / NR | NR | NR / NR | NR | NR / NR | NR | NR / NR | NR | 1 | NIM | 6 | NR | NR |
| Srinivasan 2011^75^ | CRS (5) | 1 | 42 | F | Hashimoto thyroiditis; diabetes mellitus | NR | AZA; Ig; PRED | Rituximab | 375 mg/m^2, Q1W for 4 weeks | MMF [UNK, 500 mg, BID] | NR | NR / NR | NR | NR / NR | NR | NR / NR | NR | NR / NR | NR | 4 | IM | 6 | NR | NR |
| Steier 2005^76^ | CRS (5) | 1 | 56 | M | NR | NR | AZA; CYC; HCQ | Rituximab | 210 mg, Q1W for 6 weeks | MTX [UNK, 20mg, Q1W]; PRED [PO, 7.5 mg, QD] | NR | NR / NR | NR | NR / NR | NR | NR / NR | NR | NR / NR | NR | 6 | IM | 20 | NR | NR |
| Stewart 2022^77^ | CRS (5) | 1 | 14 | F | Non-functioning micropituitary adenoma; concussion; chronic constipation; reflux; anxiety; depression; persistent daily fevers; hand, foot, and mouth disease; macrophage activation syndrome | Juvenile | CsA; Ig; METHYLPRED; MMF; PRED | Anakinra | 2-15mg/kg, BID to QD | dexamethasone [NR, 10 mg/m^2, BID]; etoposide [NR, 150 mg/m^2, BIW]; IVIG [IV, NR, NR]; PRED [PO, NR, NR] [PO, 5 mg, QD]; tofacitinib [PO, 5 mg, BID] | NR | NR / NR | NR | NR / NR | NR | NR / NR | NR | NR / NR | NR | NR | IM | NR | NR | NR |
| Su 2022^78^ | CRS (5) | 1 | 50 | M | Interstitial lung disease; macrophage activation syndrome | Adult | AZA; HCQ; Ig; METHYLPRED; mycophenolic acid; PRED | 1. Tocilizumab / 2. Rituximab | 1. (8 mg/kg, NR); 2. (1000 mg, 3 doses) | CsA [PO, 100 mg, QD]; PRED [PO, tapered down to 5 mg, QD] | NR | NR / NR | NR | NR / NR | NR | NR / NR | NR | NR / NR | NR | NR / 24 | IM / IM | 23 | NR / NR | NR / NR |
| Sukumaran 2020^79^ | CRS (5) | 1 | 16 | F | NR | NR | CYC; CS [SYSTEMIC]; MTX | Abatacept | 10 mg/kg/dose, Q2W to Q4W | CS [PO, NR, NR]; IVIG [IV, NR, NR] | NR | NR / NR | NR | NR / NR | NR | NR / NR | NR | NR / NR | NR | 12 | IM | 12 | NR | NR |
| Sullivan 2021^80^ | CRS (5) | 1 | 29 | F | NR | Adult | HCQ; MTX; PRED | Rituximab | NR | NR | NR | NR / NR | NR | NR / NR | NR | NR / NR | NR | NR / NR | NR | NR | IM | NR | NR | NR |
| Tjarnlund 2018^81^ | RCT (1b) | 9 | 51.5 | M(3); F(6) | NR | NR | AZA; CS [SYSTEMIC]; MTX | Abatacept | 500-1000mg, Q2W to Q4W | NR | Abatacept | NR / NR | NR | 11 / 5 | -54.6% | NR / NR | NR | 69.5 / 73.3 | 5.18417462482946% | 24 | IM (2); NIM (7) | 9 | Infections (14); cardiovascular events (4); tumours (3); MSK-related (3); GI upset (3); UTI (1); neuropathological (1) | NR |
| Toplak 2022^82^ | RS (2b) | 4 | 7.6 | F (4) | NR | Juvenile | METHYLPRED (4); MTX (4); CsA (2); HCQ (4); MMF (3); Ig (3); anti-TNF alpha (1); CYC (1) | Rituximab | NR | NR | NR | NR / NR | NR | NR / NR | NR | NR / NR | NR | NR / NR | NR | NR | IM (2); NIM (1); WOR (1) | NR | NR | NR |
| Touma 2008^83^ | CRS (5) | 1 | 25 | F | NR | NR | Etanercept; MTX; PRED | Rituximab | 1mg, Q2W for 2 weeks | METHYLPRED [IV, 1mg/ day, 3 days] | NR | NR / NR | NR | NR / NR | NR | NR / NR | NR | NR / NR | NR | 4 | IM | 1 | NR | NR |
| Tzaribachev 2009^84^ | CAS (4) | 1 | 14 | F | NR | NR | CsA; METHYLPRED; PRED | Rituximab | 375 mg/m^2, 4 doses | MTX [SC, 20mg/m^2, Q1W] | NR | NR / NR | NR | NR / NR | NR | NR / NR | NR | NR / NR | NR | NR | NIM | 2 | NR | NR |
| Valls 2022^85^ | CRS (5) | 1 | 48 | F | Ovarian cancer | Adult | CS [SYSTEMIC]; CYC; Ig | Rituximab | NR, Q8W | Doxycycline [PO, NR, NR]; gemcitabine [PO, NR, NR]; HCQ [PO, NR, NR]; pembrolizumab [IV, NR, NR]; PRED [PO, NR, QD]; tofacitinib [PO, NR, NR] | NR | NR / NR | NR | NR / NR | NR | NR / NR | NR | NR / NR | NR | 15 | IM | 7 | NR | NR |
| VanEngelen 2003^86^ | CAS (4) | 1 | 50 | F | Hypertension | NR | NR | Infliximab | 10 mg/kg, Q2W | NR | NR | NR / NR | NR | NR / NR | NR | NR / NR | NR | NR / NR | NR | 2 | IM | 12 | NR | NR |
| VanEngelen 2008^87^ | CoH (2b) | 4 | 48 | F | NR | NR | NR | Infliximab | 10 mg/kg, Q2W to Q4W to Q8W | Folic acid [PO, 5mg, Q1W]; MTX [UNK, 15mg, Q1W] | NR | NR / NR | NR | NR / NR | NR | NR / NR | NR | NR / NR | NR | 6 | IM (2); NIM (2) | 1.5 | Malignancy (1); autoimmune skin rash (1) | NR |
| Wendel 2019^88^ | CAS (4) | 1 | 55 | F | NR | NR | AZA; HCQ; MMF; tacrolimus | Rituximab | NR | NR | NR | NR / NR | NR | NR / NR | NR | NR / NR | NR | NR / NR | NR | NR | NIM | 7 | NR | NR |
| Xie 2020^89^ | CRS (5) | 1 | 24 | F | NR | NR | CsA; HCQ; Ig; MTX; mycophenolate; rituximab | Adalimumab | 40 mg, Q1W | NR | NR | NR / NR | NR | NR / NR | NR | NR / NR | NR | NR / NR | NR | 120 | IM | 30 | NR | NR |
| Yamada-Kanazawa 2019^90^ | CRS (5) | 1 | 44 | F | NR | NR | MTX | Infliximab | 5mg/kg, NR | NR | NR | NR / NR | NR | NR / NR | NR | NR / NR | NR | NR / NR | NR | 6 | IM | 12 | NR | NR |
| Zhang 2021^91^ | CAS (4) | 1 | 43 | M(3); F(3) | NR | NR | CsA; Ig; METHYLPRED; PRED | Tocilizumab | 480 mg, Q1W | CYC [PO, 0.1g, QD]; PRED [PO, 60mg, QD]; tacrolimus [PO, 1mg, QD] | NR | NR / NR | NR | NR / NR | NR | NR / NR | NR | NR / NR | NR | 4 | IM | 12 | CMV infection; liver dysfunction | NR |

References:

1. Aeschlimann FA, Saire E, Neven B, et al. A child with severe juvenile dermatomyositis treated with ruxolitinib. *Brain*. 2018;141(11):e80. doi:[10.1093/brain/awy255](https://doi.org/10.1093/brain/awy255)
2. Aggarwal R, Loganathan P, Koontz D, Qi Z, Oddis CV, Reed AM. Cutaneous improvement in refractory adult and juvenile dermatomyositis after treatment with rituximab. *Rheumatology (United Kingdom)*. 2017;56(2):247-254. doi:[10.1093/rheumatology/kew396](https://doi.org/10.1093/rheumatology/kew396)
3. Albakri A.M., Subki A.H., Albeity A., Halabi H. Dermatomyositis Flare After a COVID-19 Infection Successfully Treated with Rituximab: A Case Report and Literature Review. *Journal of Inflammation Research*. 2022;15((Albakri, Subki, Albeity, Halabi) Department of Medicine, King Faisal Specialist Hospital and Research Center, Jeddah, Saudi Arabia):6047-6053. doi:[10.2147/JIR.S369477](https://doi.org/10.2147/JIR.S369477)
4. Amato A. A randomized, pilot trial of etanercept in dermatomyositis. *Annals of Neurology*. 2011;70(3):427-436. doi:[10.1002/ana.22477](https://doi.org/10.1002/ana.22477)
5. Ang PS, Ezenwa E, Ko K, Hoffman MD. Refractory dermatomyositis responsive to anifrolumab. *JAAD Case Rep*. 2023;43:27-29. Published 2023 Nov 7. doi:10.1016/j.jdcr.2023.10.023
6. Arabshahi B, Silverman RA, Jones OY, Rider LG. Abatacept and Sodium Thiosulfate for Treatment of Recalcitrant Juvenile Dermatomyositis Complicated by Ulceration and Calcinosis. *Journal of Pediatrics*. ((Arabshahi) Division of Pediatric Rheumatology, Department of Pediatrics, Inova Fairfax Hospital for Children, Fairfax, VA(Silverman) Department of Pediatrics, Georgetown University, Washington, DC(Jones) Division of Pediatric Rheumatology, Department of). doi:[10.1016/j.jpeds.2011.11.057](https://doi.org/10.1016/j.jpeds.2011.11.057)
7. Argobi Y, Fedeles F, Schoenfeld SR. A case of Wong-type dermatomyositis treated with rituximab and IVIG. *Australasian Journal of Dermatology*. 2021;62(1):e86-e87. doi:[10.1111/ajd.13393](https://doi.org/10.1111/ajd.13393)
8. Bader-Meunier B, Quartier P, Decaluwe H, et al. Safety and efficacy of rituximab in severe juvenile dermatomyositis: Results from 9 patients from the French autoimmunity and rituximab registry. *Journal of Rheumatology*. 2011;38(7):1436-1440. doi:[10.3899/jrheum.101321](https://doi.org/10.3899/jrheum.101321)
9. Castano-Amores C, Nieto-Gomez P. Cerebral toxoplasmosis associated with treatment with rituximab, azathioprine and prednisone for dermatomyositis. *British Journal of Clinical Pharmacology*. 2021;87(3):1525-1528. doi:[10.1111/bcp.14445](https://doi.org/10.1111/bcp.14445)
10. Chalhoub N, Georgescu C, Altorok N. Mycobacterium Avium Complex Septic Arthritis in a Patient Treated by Infliximab. *American Journal of Therapeutics*. 2016;23(5):e1222-e1225. doi:[10.1097/MJT.0000000000000318](https://doi.org/10.1097/MJT.0000000000000318)
11. Chen D, Wang XB, Zhou Y, Zhu XC. Efficacy of infliximab in the treatment for dermatomyositis with acute interstitial pneumonia: A study of fourteen cases and literature review. *Rheumatology International*. 2013;33(10):2455-2458. doi:[10.1007/s00296-012-2653-4](https://doi.org/10.1007/s00296-012-2653-4)
12. Choi KH, Yoo WH. Necrotizing fasciitis in a patient treated with etanercept for dermatomyositis. *Rheumatology International*. 2009;29(4):463-466. doi:[10.1007/s00296-008-0695-4](https://doi.org/10.1007/s00296-008-0695-4)
13. Chung L, Genovese MC, Fiorentino DF. A pilot trial of rituximab in the treatment of patients with dermatomyositis. *Archives of Dermatology*. 2007;143(6):763-767. doi:[10.1001/archderm.143.6.763](https://doi.org/10.1001/archderm.143.6.763)
14. Clottu A, Chizzolini C, Laffitte E, Prins C. Response of mucocutaneous lesions to rituximab in a case of melanoma differentiation antigen 5-related dermatomyositis. *Dermatology*. 2012;225(4):376-380. doi:[10.1159/000346573](https://doi.org/10.1159/000346573)
15. Cooper MA, Willingham DL, Brown DE, French AR, Shih FF, White AJ. Rituximab for the treatment of juvenile dermatomyositis: A report of four pediatric patients. *Arthritis and Rheumatism*. 2007;56(9):3107-3111. doi:[10.1002/art.22856](https://doi.org/10.1002/art.22856)
16. Crespo Cruz A, del Boz J, Romero Gomez C. Good Response to Tofacitinib in Refractory Amyopathic Dermatomyositis. *Actas Dermo-Sifiliograficas*. 2021;112(4):374-376. doi:[10.1016/j.adengl.2021.01.034](https://doi.org/10.1016/j.adengl.2021.01.034)
17. Curiel R.V., Nguyen W., Mamyrova G., et al. Improvement in Disease Activity in Refractory Juvenile Dermatomyositis Following Abatacept Therapy. *Arthritis & rheumatology (Hoboken, NJ)*. 2023;((Curiel, Nguyen, Mamyrova, Jones, Kim, Jones, Rider) Division of Rheumatology, Department of Medicine, George Washington University School of Medicine and Health Sciences, WA, United States(Ehrlich) Department of Dermatology, George Washington University). doi:[10.1002/art.42450](https://doi.org/10.1002/art.42450)
18. Day W, Gabriel C, Aguiar CL, et al. Juvenile dermatomyositis resembling late-stage Degos disease with gastrointestinal perforations successfully treated with combination of cyclophosphamide and rituximab: case-based review. *Rheumatology International*. 2020;40(11):1883-1890. doi:[10.1007/s00296-019-04495-2](https://doi.org/10.1007/s00296-019-04495-2)
19. De Lorenzo R, Cavalli S, Bonomi F, et al. Begelomab for severe refractory dermatomyositis: A case report. *Medicine*. 2021;100(9):e24372. doi:[10.1097/MD.0000000000024372](https://doi.org/10.1097/MD.0000000000024372)
20. de Souza FHC, Miossi R, de Moraes JCB, Bonfa E, Shinjo SK. Favorable rituximab response in patients with refractory idiopathic inflammatory myopathies. *Advances in rheumatology (London, England)*. 2018;58(1):31. doi:[10.1186/s42358-018-0030-z](https://doi.org/10.1186/s42358-018-0030-z)
21. Dinh HV, McCormack C, Hall S, Prince HM. Rituximab for the treatment of the skin manifestations of dermatomyositis: A report of 3 cases. *Journal of the American Academy of Dermatology*. 2007;56(1):148-153. doi:[10.1016/j.jaad.2006.05.068](https://doi.org/10.1016/j.jaad.2006.05.068)
22. Dold S, Justiniano ME, Marquez J, Espinoza LR. Treatment of early and refractory dermatomyositis with infliximab: A report of two cases. *Clinical Rheumatology*. 2007;26(7):1186-1188. doi:[10.1007/s10067-006-0325-z](https://doi.org/10.1007/s10067-006-0325-z)
23. Efthimiou P, Schwartzman S, Kagen LJ. Possible role for tumour necrosis factor inhibitors in the treatment of resistant dermatomyositis and polymyositis: A retrospective study of eight patients. *Annals of the Rheumatic Diseases*. 2006;65(9):1233-1236. doi:[10.1136/ard.2005.048744](https://doi.org/10.1136/ard.2005.048744)
24. Fernandez RR, Rubio JLC, Cano DS, Centeno NO, Moreno JAS. Rituximab in the treatment of dermatomyositis and other inflammatory myopathies. A report of 4 cases and review of the literature. *Clinical and Experimental Rheumatology*. 2009;27(6):1009-1016.
25. Fetter T, Rios GC, Niebel D, Bieber T, Wenzel J. Unexpected Hair Regrowth in a Patient with Longstanding Alopecia Universalis During Treatment of Recalcitrant Dermatomyositis with the Janus Kinase Inhibitor Ruxolitinib. *Acta dermato-venereologica*. 2020;100(10):adv00144. doi:[10.2340/00015555-3481](https://doi.org/10.2340/00015555-3481)
26. Ge Y, Li S, Tian X, He L, Lu X, Wang G. Anti-melanoma differentiation-associated gene 5 (MDA5) antibody-positive dermatomyositis responds to rituximab therapy. *Clinical Rheumatology*. 2021;40(6):2311-2317. doi:[10.1007/s10067-020-05530-5](https://doi.org/10.1007/s10067-020-05530-5)
27. Groh M, Rogowska K, Monsarrat O, Denoel A, Blanche P, Guillevin L. Interleukin-1 receptor antagonist for refractory anti-MDA5 clinically amyopathic dermatomyopathy. *Clinical and experimental rheumatology*. 2015;33(6):904-905.
28. Grundtman C, Barbasso Helmers S, Lundberg IE, et al. A high incidence of disease flares in an open pilot study of infliximab in patients with refractory inflammatory myopathies. *Annals of the Rheumatic Diseases*. 2008;67(12):1670-1677. doi:[10.1136/ard.2007.077974](https://doi.org/10.1136/ard.2007.077974)
29. Gullapalli K, Goldzweig O, Nanda K, Chekka R, Berry S, Bukulmez H. Juvenile Dermatomyositis (JDM) Complicated by Thrombotic Thrombocytopenic Purpura (TTP) and Purtscher’s Retinopathy Responsive to Rituximab: Case Report and Literature Review. *Frontiers in Pediatrics*. 2020;8((Gullapalli) Internal Medicine, Sparrow Hospital, Michigan State University, Lansing, MI, United States(Goldzweig) Kaplan Medical Center, Rehovot, Israel(Nanda) Seattle Children’s Hospital, Seattle, WA, United States(Chekka) Pediatric Care Center, Erie, P):436. doi:[10.3389/fped.2020.00436](https://doi.org/10.3389/fped.2020.00436)
30. Haroon M, Devlin J. Rituximab as a first-line agent for the treatment of dermatomyositis. *Rheumatology International*. 2012;32(6):1783-1784. doi:[10.1007/s00296-010-1458-6](https://doi.org/10.1007/s00296-010-1458-6)
31. Hassan N, Davies EJ, Faber BG, Gunawardena H. Infliximab in a patient with treatment-resistant anti-SAE dermatomyositis. *Rheumatology (United Kingdom)*. 2021;60(5):E156-E158. doi:[10.1093/rheumatology/keaa698](https://doi.org/10.1093/rheumatology/keaa698)
32. Hisanaga J, Kotani T, Fujiki Y, Yoshida S, Takeuchi T, Makino S. Successful multi-target therapy including rituximab and mycophenolate mofetil in anti-melanoma differentiation-associated gene 5 antibody-positive rapidly progressive interstitial lung disease with clinically amyopathic dermatomyositis. *International Journal of Rheumatic Diseases*. 2017;20(12):2182-2185. doi:[10.1111/1756-185X.13136](https://doi.org/10.1111/1756-185X.13136)
33. Holzer U, Kuemmerle-Deschner J, Well C, et al. Successful autologous stem cell transplantation in two patients with juvenile dermatomyositis. *Scandinavian Journal of Rheumatology*. 2010;39(1):88-92. doi:[10.3109/03009740903096622](https://doi.org/10.3109/03009740903096622)
34. Huang K, Shojania K, Yeung J, et al. Clinical spectrum and therapeutics in Canadian patients with anti-melanoma differentiation-associated gene 5 (MDA5)-positive dermatomyositis: a case-based review. *Rheumatology International*. 2019;39(11):1971-1981. doi:[10.1007/s00296-019-04398-2](https://doi.org/10.1007/s00296-019-04398-2)
35. Iannone F, Scioscia C, Falappone PCF, Covelli M, Lapadula G. Use of etanercept in the treatment of dermatomyositis: A case series. *Journal of Rheumatology*. 2006;33(9):1802-1804.
36. Jin P., Yang C., Bai J., Dong L., Zhi L. Successfully treatment with Dupilumab for systemic contact dermatitis following hair dye in a patient with dermatomyositis. *Journal of Cosmetic Dermatology*. 2022;21(11):6468-6469. doi:[10.1111/jocd.15112](https://doi.org/10.1111/jocd.15112)
37. Jois R, Srinivasan P, Vasudevan N, Mehta R. Resistant dermatomyositis complicated by tubercular myositis and successfully treated with rituximab. *Neurology India*. 2011;59(2):306-307. doi:[10.4103/0028-3886.79163](https://doi.org/10.4103/0028-3886.79163)
38. Joshi N, Nautiyal A, Davies PG. Successful use of rituximab in recalcitrant skin predominant dermatomyositis. *Journal of Clinical Rheumatology*. 2011;17(2):111-112. doi:[10.1097/RHU.0b013e31821072b7](https://doi.org/10.1097/RHU.0b013e31821072b7)
39. Kaposztas Z, Kahan BD, Etheridge WB. Case Report: Successful Treatment of Posttransplant Lymphoproliferative Disorder and Quiescence of Dermatomyositis With Rituximab and Sirolimus. *Transplantation Proceedings*. 2008;40(5):1744-1746. doi:[10.1016/j.transproceed.2007.11.072](https://doi.org/10.1016/j.transproceed.2007.11.072)
40. Komiya Y, Saito T, Mizoguchi F, Kohsaka H. Hemophagocytic syndrome complicated with dermatomyositis controlled successfully with infliximab and conventional therapies. *Internal Medicine*. 2017;56(23):3237-3241. doi:[10.2169/internalmedicine.7966-16](https://doi.org/10.2169/internalmedicine.7966-16)
41. Kondo M, Murakawa Y, Matsumura T, et al. A case of overlap syndrome successfully treated with tocilizumab: a hopeful treatment strategy for refractory dermatomyositis? *Rheumatology (Oxford, England)*. 2014;53(10):1907-1908. doi:[10.1093/rheumatology/keu234](https://doi.org/10.1093/rheumatology/keu234)
42. Kuye IO, Smith GP. The use of rituximab in the management of refractory dermatomyositis. *Journal of Drugs in Dermatology*. 2017;16(2):162-166.
43. Lam SC, Yuen HKL. Unilateral Eyelid Swelling as a Sign of Antimelanoma Differentiation-Associated Gene 5 (Anti-MDA5)-Antibody-Positive Dermatomyositis. *Ophthalmic Plastic and Reconstructive Surgery*. 2018;34(6):e209-e211. doi:[10.1097/IOP.0000000000001258](https://doi.org/10.1097/IOP.0000000000001258)
44. Levine TD. Rituximab in the treatment of dermatomyositis: An open-label pilot study. *Arthritis and Rheumatism*. 2005;52(2):601-607. doi:[10.1002/art.20849](https://doi.org/10.1002/art.20849)
45. Li S, Sun Y, Shao C, Huang H, Xu K. Diffuse large B-cell lymphoma in a patient with dermatomyositis-associated interstitial lung disease: A case report. *Thoracic Cancer*. 2019;10(10):2035-2039. doi:[10.1111/1759-7714.13171](https://doi.org/10.1111/1759-7714.13171)
46. Lim DH, So MW, Kim YM, et al. Clinically amyopathic dermatomyositis presenting with isolated facial edema complicated by acute respiratory failure: a case report. *BMC Musculoskeletal Disorders*. 2021;22(1):117. doi:[10.1186/s12891-021-03996-1](https://doi.org/10.1186/s12891-021-03996-1)
47. Lu Z, Chen Y, Xue J, Liu L. NXP2-positive dermatomyositis complicated with refractory skin edema: Successful treatment with tocilizumab. *Dermatologic Therapy*. 2021;34(1):e14712. doi:[10.1111/dth.14712](https://doi.org/10.1111/dth.14712)
48. Lyakhovitsky A, Warshavsky K, Rozner L, Drousiotis T, Baum S, Barzilai A. Dermatomyositis-lupuslike syndrome overlap under treatment with etanercept for rheumatoid arthritis. *JAAD Case Reports*. 2020;6(8):758-760. doi:[10.1016/j.jdcr.2020.06.014](https://doi.org/10.1016/j.jdcr.2020.06.014)
49. Majmudar S, Hall HA, Zimmermann B. Treatment of adult inflammatory myositis with rituximab: An emerging therapy for refractory patients. *Journal of Clinical Rheumatology*. 2009;15(7):338-340. doi:[10.1097/RHU.0b013e3181bb8e70](https://doi.org/10.1097/RHU.0b013e3181bb8e70)
50. Montoya CL, Gonzalez ML, Ospina FE, Tobon GJ. A rare case of amyopathic juvenile dermatomyositis associated with psoriasis successfully treated with ustekinumab. *Journal of Clinical Rheumatology*. 2017;23(2):129-130. doi:[10.1097/RHU.0000000000000430](https://doi.org/10.1097/RHU.0000000000000430)
51. Munoz-Beamud F, Isenberg DA. Rituximab as an effective alternative therapy in refractory idiopathic inflammatory myopathies. *Clinical and Experimental Rheumatology*. 2013;31(6):896-903.
52. Navarro-Navarro I, Jimenez-Gallo D, Linares-Barrios M, Rodriguez-Mateos ME, Rodriguez-Hernandez C. Treatment of refractory anti-NXP2 and anti-TIF1gamma dermatomyositis with tofacitinib. *JDDG - Journal of the German Society of Dermatology*. 2021;19(3):443-447. doi:[10.1111/ddg.14276](https://doi.org/10.1111/ddg.14276)
53. Neri P., Aljneibi S., Pichi F. Rescue Treatment with Infliximab for a Bilateral, Severe, Sight Threatening Frosted Branch Angiitis Associated with Concomitant Acute Onset of Presumed Dermatomyositis. *Ocular Immunology and Inflammation*. 2022;((Neri, Aljneibi, Pichi) The Eye Institute, Cleveland Clinic Abu Dhabi, Abu Dhabi, United Arab Emirates(Neri, Pichi) Cleveland Clinic Lerner College of Medicine, Case Western Reserve University (USA), Cleveland, OH, United States(Neri) College of Medicine). doi:[10.1080/09273948.2022.2057333](https://doi.org/10.1080/09273948.2022.2057333)
54. Noss EH, Hausner-Sypek DL, Weinblatt ME. Rituximab as therapy for refractory polymyositis and dermatomyositis. *Journal of Rheumatology*. 2006;33(5):1021-1026.
55. O’Brien MA, Hsu S, Shevchenko A, Quartey A. Dupilumab Failure in Treating Dermatomyositis-Associated Pruritus. *Cureus*. 2022;14(8):e28270. doi:[10.7759/cureus.28270](https://doi.org/10.7759/cureus.28270)
56. Oddis C.V., Rockette H.E., Zhu L., et al. Randomized Trial of Tocilizumab in the Treatment of Refractory Adult Polymyositis and Dermatomyositis. *ACR Open Rheumatology*. 2022;4(11):983-990. doi:[10.1002/acr2.11493](https://doi.org/10.1002/acr2.11493)
57. Oddis CV, Aggarwal R, Levesque MC, et al. Rituximab in the treatment of refractory adult and juvenile dermatomyositis and adult polymyositis: A randomized, placebo-phase trial. *Arthritis and Rheumatism*. 2013;65(2):314-324. doi:[10.1002/art.37754](https://doi.org/10.1002/art.37754)
58. Ogawa Y, Kishida D, Shimojima Y, Hayashi K, Sekijima Y. Effective Administration of Rituximab in Anti-MDA5 Antibody-Positive Dermatomyositis with Rapidly Progressive Interstitial Lung Disease and Refractory Cutaneous Involvement: A Case Report and Literature Review. *Case reports in rheumatology*. 2017;2017(101585353):5386797. doi:[10.1155/2017/5386797](https://doi.org/10.1155/2017/5386797)
59. Paik JJ, Christopher-Stine L. A case of refractory dermatomyositis responsive to tofacitinib. *Seminars in Arthritis and Rheumatism*. 2017;46(4):e19. doi:[10.1016/j.semarthrit.2016.08.009](https://doi.org/10.1016/j.semarthrit.2016.08.009)
60. Pandya R, Lim D, Kleitsch J, Werth VP. Overlap of dermatomyositis and cutaneous lupus erythematosus: A case series. *JAAD Case Rep*. 2023;42:95-101. Published 2023 Oct 14. doi:10.1016/j.jdcr.2023.10.002
61. Park JK, Ahn DS, Jeon HS, Yoo WH, Yoo HG. Successful treatment for conventional treatment-resistant dermatomyositis-associated interstitial lung disease with adalimumab. *Rheumatology International*. 2012;32(11):3587-3590. doi:[10.1007/s00296-011-2220-4](https://doi.org/10.1007/s00296-011-2220-4)
62. Rider LG, Yip AL, Volochayev R, et al. Novel assessment tools to evaluate clinical and laboratory responses in a subset of patients enrolled in the Rituximab in Myositis trial. *Clinical and Experimental Rheumatology*. 2014;32(5):689-696.
63. Riley P, Mccann LJ, Maillard SM, Woo P, Pilkington CA, Murray KJ. Effectiveness of infliximab in the treatment of refractory juvenile dermatomyositis with calcinosis. *Rheumatology*. 2008;47(6):877-880. doi:[10.1093/rheumatology/ken074](https://doi.org/10.1093/rheumatology/ken074)
64. Rodziewicz M, Kiely P. The successful use of subcutaneous abatacept in refractory anti- human transcriptional intermediary factor 1-gamma dermatomyositis skin and oesphagopharyngeal disease. *Rheumatology (United Kingdom)*. 2018;57(10):1866-1867. doi:[10.1093/rheumatology/key146](https://doi.org/10.1093/rheumatology/key146)
65. Rouster-Stevens KA, Ferguson L, Morgan G, Huang CC, Pachman LM. Pilot study of etanercept in patients with refractory juvenile dermatomyositis. *Arthritis Care and Research*. 2014;66(5):783-787. doi:[10.1002/acr.22198](https://doi.org/10.1002/acr.22198)
66. Sabbagh S, De Jesus AA, Goldbach-Mansky R, et al. Treatment of anti-MDA5 autoantibody-positive juvenile dermatomyositis using tofacitinib. *Brain*. 2019;142(11):E59. doi:[10.1093/brain/awz293](https://doi.org/10.1093/brain/awz293)
67. Sallum AME, Silva MFC, Michelin CM, et al. Penile and scrotum swelling in juvenile dermatomyositis. *Acta Reumatologica Portuguesa*. 2011;36(2):176-179.
68. Sanchez-Ramon S, Ravell JC, Rodriguez-Mahou M, et al. Long-term remission of severe refractory dermatopolymyositis with a weekly-scheme of immunoglobulin followed by rituximab therapy. *Rheumatology International*. 2010;30(6):817-819. doi:[10.1007/s00296-009-1000-x](https://doi.org/10.1007/s00296-009-1000-x)
69. Schiffenbauer A, Faghihi-Kashani S, Miller FW, et al. A randomized, double-blind, placebo-controlled trial of infliximab in refractory polymyositis and dermatomyositis. *Seminars in Arthritis and Rheumatism*. 2018;47(6):858-864. doi:[10.1016/j.semarthrit.2017.10.010](https://doi.org/10.1016/j.semarthrit.2017.10.010)
70. Schildt EE, De Ranieri D. Anasarca as the presenting symptom of juvenile dermatomyositis: a case series. *Pediatric Rheumatology*. 2021;19(1):120. doi:[10.1186/s12969-021-00604-3](https://doi.org/10.1186/s12969-021-00604-3)
71. Shaw KS, Reusch DB, Castillo RL, et al. Rapid Improvement in Recalcitrant Cutaneous Juvenile Dermatomyositis With Anifrolumab Treatment. *JAMA Dermatol.* 2024;160(2):237-238. doi:10.1001/jamadermatol.2023.4744
72. Simou S, Papadopoulou C, Pilkington CA, et al. Retrospective analysis of infliximab and adalimumab treatment in a large cohort of juvenile dermatomyositis patients. *Arthritis Research and Therapy*. 2020;22(1):79. doi:[10.1186/s13075-020-02164-5](https://doi.org/10.1186/s13075-020-02164-5)
73. Sozeri B, Demir F. A striking treatment option for recalcitrant calcinosis in juvenile dermatomyositis: tofacitinib citrate. *Rheumatology (United Kingdom)*. 2020;59(12):E140-E141. doi:[10.1093/rheumatology/keaa360](https://doi.org/10.1093/rheumatology/keaa360)
74. Speth F, Haas JP, Hinze CH. Treatment with high-dose recombinant human hyaluronidase-facilitated subcutaneous immune globulins in patients with juvenile dermatomyositis who are intolerant to intravenous immune globulins: A report of 5 cases. *Pediatric Rheumatology*. 2016;14(1):52. doi:[10.1186/s12969-016-0112-6](https://doi.org/10.1186/s12969-016-0112-6)
75. Srinivasan J, Parziale N, Kovacs SC, Thomas CB. Rituximab and mycophenolate combination therapy in refractory dermatomyositis with multiple autoimmune disorders. *Journal of Clinical Neuromuscular Disease*. 2011;13(2):63-67. doi:[10.1097/CND.0b013e318221259d](https://doi.org/10.1097/CND.0b013e318221259d)
76. Steier J, Gruber B, Chiappetta N. Rituximab in the treatment of refractory dermatomyositis. *Journal of Clinical Rheumatology*. 2005;11(5):264-266. doi:[10.1097/01.rhu.0000182155.08982.60](https://doi.org/10.1097/01.rhu.0000182155.08982.60)
77. Stewart J.A., Price T., Moser S., Mullikin D., Bryan A. Refractory Macrophage Activation Syndrome Secondary to anti-mda 5 Antibody Positive Juvenile Dermatomyositis. *Pediatrics*. 2022;149. <https://publications.aap.org/pediatrics/article/149/1MeetingAbstractsFebruary2022/981/186362/Refractory-Macrophage-Activation-Syndrome>
78. Su C.-F., Liao H.-T., Tsai C.-Y. Tocilizumab and rituximab for anti-MDA-5 positive amyopathic dermatomyositis complicated with macrophage activation syndrome and progressive fibrosing interstitial lung disease. *Scandinavian Journal of Rheumatology*. 2022;51(2):166-168. doi:[10.1080/03009742.2021.1972519](https://doi.org/10.1080/03009742.2021.1972519)
79. Sukumaran S, Vijayan V. Abatacept in the Treatment of Juvenile Dermatomyositis-Associated Calcifications in a 16-Year-Old Girl. *Case reports in rheumatology*. 2020;2020(101585353):4073879. doi:[10.1155/2020/4073879](https://doi.org/10.1155/2020/4073879)
80. Sullivan T., Hildebrand B.A. Dermatomyositis: A Refractory Case in an Uninsured Patient. *Journal of Clinical Rheumatology*. 2021;27(8):S694-S696. doi:[10.1097/RHU.0000000000001588](https://doi.org/10.1097/RHU.0000000000001588)
81. Tjarnlund A, Tang Q, Wick C, et al. Abatacept in the treatment of adult dermatomyositis and polymyositis: A randomised, phase IIb treatment delayed-start trial. *Annals of the Rheumatic Diseases*. 2018;77(1):55-62. doi:[10.1136/annrheumdis-2017-211751](https://doi.org/10.1136/annrheumdis-2017-211751)
82. Toplak N., Pimpale Chavan P., Rosina S., et al. Is Anti-NXP2 Autoantibody a Risk Factor for Calcinosis and Poor Outcome in Juvenile Dermatomyositis Patients? Case Series. *Frontiers in Pediatrics*. 2022;9((Toplak) Department of Allergology, Rheumatology and Clinical Immunology, Faculty of Medicine, University Children’s Hospital, University Medical Centre, Ljubljana, Slovenia(Pimpale Chavan, Khubchandani) Pediatric Rheumatology, NH SRCC Children’s Hospital):810785. doi:[10.3389/fped.2021.810785](https://doi.org/10.3389/fped.2021.810785)
83. Touma Z, Arayssi T, Kibbi L, Masri AF. Successful treatment of cardiac involvement in dermatomyositis with rituximab. *Joint Bone Spine*. 2008;75(3):334-337. doi:[10.1016/j.jbspin.2007.05.011](https://doi.org/10.1016/j.jbspin.2007.05.011)
84. Tzaribachev N, Kuemmerle-Deschner JB, Koetter I, Schedel J. Rituximab for the treatment of refractory pediatric autoimmune diseases: A case series. *Cases Journal*. 2009;2(8):6609. doi:[10.4076/1757-1626-2-6609](https://doi.org/10.4076/1757-1626-2-6609)
85. Valls M.L., Kase A.M., Patel R., Wang B., Aggarwal R., Colon-Otero G. Complete response to pembrolizumab in a patient with dermatomyositis and MMR deficient ovarian cancer: A case report. *Gynecologic Oncology Reports*. 2022;41((Valls) Ponce Health Sciences University, Ponce, PR(Kase, Colon-Otero) Division of Hematology/Oncology(Wang) Division of Rheumatology, Mayo Clinic, Jacksonville, FL(Patel) Florida Cancer Specialists, Jupiter, FL(Aggarwal) Division of Rheumatology, Univers):101010. doi:[10.1016/j.gore.2022.101010](https://doi.org/10.1016/j.gore.2022.101010)
86. Van Engelen BGM, Van Den Hoogen FHJ, Barrera P, et al. Successful treatment of dermatomyositis and polymyositis with anti-tumor-necrosis-factor-alpha: Preliminary observations. *European Neurology*. 2003;50(1):10-15. doi:[10.1159/000070852](https://doi.org/10.1159/000070852)
87. Van Engelen BGM, Van Den Hoogen FHJ, De Bleecker JL, et al. Open-label trial of anti-TNF-alpha in dermato- and polymyositis treated concomitantly with methotrexate. *European Neurology*. 2008;59(3-4):159-163. doi:[10.1159/000114036](https://doi.org/10.1159/000114036)
88. Wendel S, Venhoff N, Rizzi M, et al. Successful treatment of extensive calcifications and acute pulmonary involvement in dermatomyositis with the Janus-Kinase inhibitor tofacitinib - A report of two cases. *Journal of Autoimmunity*. 2019;100((Wendel, Venhoff, Rizzi, Voll, Thiel) Department of Internal Medicine, Clinic for Rheumatology and Clinical Immunology, Medical Center-University of Freiburg, Faculty of Medicine, Hugstetter Str. 55, Freiburg 79106, Germany(Frye) Department of Internal):131-136. doi:[10.1016/j.jaut.2019.03.003](https://doi.org/10.1016/j.jaut.2019.03.003)
89. Xie F, Williams P, Batchelor R, Downs A, Haigh R. Successful treatment of dermatomyositis and associated calcinosis with adalimumab. *Clinical and Experimental Dermatology*. 2020;45(7):945-949. doi:[10.1111/ced.14325](https://doi.org/10.1111/ced.14325)
90. Yamada-Kanazawa S, Kajihara I, Kobayashi A, Watanabe C, Ihn H. Infliximab improved the refractory cutaneous involvement in a patient with dermatomyositis. *Dermatologic Therapy*. 2019;32(3):e12859. doi:[10.1111/dth.12859](https://doi.org/10.1111/dth.12859)
91. Zhang X, Zhou S, Wu C, et al. Tocilizumab for refractory rapidly progressive interstitial lung disease related to anti-MDA5-positive dermatomyositis. *Rheumatology (United Kingdom)*. 2021;60(7):E227-E228. doi:[10.1093/rheumatology/keaa906](https://doi.org/10.1093/rheumatology/keaa906)
