# Supplementary material for: Management of Cutaneous Dermatomyositis With Systemic Biologic Therapies: A Systematic Review
Source: J Cutan Med Surg. 2024 Jul 26;28(5):490–1. doi: 10.1177/12034754241265717 (PMC11528838; doi:10.1177/12034754241265717)
Supplement: sj-docx-4-cms-10.1177_12034754241265717 – Supplemental material for Management of Cutaneous Dermatomyositis With Systemic Biologic Therapies: A Systematic Review [file sj-docx-4-cms-10.1177_12034754241265717.docx]

**Supplemental Table 3.** Outcomes of systemic biologic use for dermatomyositis. Abbreviations: CDASI, Cutaneous Dermatomyositis Area and Severity Index; DAS, Disease Activity Score; IM, improvement; MMT, Manual Muscle Testing; N, no, NIM, no improvement; NR: none reported; UTI, urinary tract infection; VAS, Visual Analogue Scale; WoR, worsening; Y, yes.

| **Biologic agent (%, n)** | **Level of evidence (n/N)** | **Mean change in skin-related metrics from baseline, % (n/N)** | **Mean change in MMT from baseline (n/N)** | **Treatment skin outcome based on reported general impression: (%, n/N)** | **Treatment duration in weeks (n/N)** | **Adverse events (n)** | **Mean follow-up period, months (n/N)** |
| --- | --- | --- | --- | --- | --- | --- | --- |
| Rituximab (68.3%, 402/589) | Case report (31/54) Case series (13/54) Retrospective study (6/54)  Randomized controlled trial (2/54)  Prospective cohort study (2/54) | CDASI: -19.4% (18/402)  Skin-DAS: 0% (10/402)  Skin-VAS: -47% (120/402) | 17.7% (38/402) | IM (80.1%, 322/402) | 42.6 (264/322) | UTI (30/402); infections requiring oral antibiotics (6/402); hypersensitivity reactions (3/402); joint pain/swelling (3/402); altered mentality (1/402); aspiration pneumonia (1/402); cerebral toxoplasmosis (1/402); malignancy (colon and lung) (1/402); papular necrosis (1/402); enterococcal sepsis (1/402); seborrheic dermatitis (1/402) | 7 (303/322) |
|  |  |  |  | NIM (19.7%, 79/402) | 39.3 (39/79) |  | 10.9 (62/79) |
|  |  |  |  | WoR (0.2%, 1/402) | NR |  | NR |
| Infliximab (16%, 94/589) | Case report (6/14)  Case series (4/14)  Retrospective study (2/14)  Randomized controlled trial (1/14)  Prospective cohort study (1/14) | Skin-DAS: -75% (39/94) | 99.3% (5/94) | IM (69.1%, 65/94) | 18.8 (49/65) | Hypersensitivity reaction (9/94); infection (2/94); autoimmune skin rash (1/94); malignancy (1/94); mycobacterial infection (1/94) | 14 (54/65) |
|  |  |  |  | NIM (30.9%, 29/94) | 12 (20/27) |  | 9 (23/27) |
| Etanercept (5.6%, 33/589) | Case report (2/5)  Randomized controlled trial (1/5)  Retrospective study (1/5)  Case series (1/5) | CDASI: -26% (11/33) | 4.9% (11/33) | IM (54.5%, 18/33) | 28.7 (12/18) | Cardiac irregularity (1/33); dermatomyositis-lupus like overlap eruption (1/33); elevated ANA (1/33); lupus-like eruption (1/33); nasal congestion (1/33); necrotizing fasciitis (1/33); recurrent esophagitis; splenic tumour/ascites (1/33) | 9.6 (12/18) |
|  |  |  |  | NIM (27.3%, 9/33) | 15.9 (8/9) |  | 3.8 (8/9) |
|  |  |  |  | WoR (18.2%, 6/33) | 45.3 (6/6) |  | 6 (1/6) |
| Abatacept (3.6%, 21/589) | Case report (2/4)  Randomized controlled trial (1/4)  Prospective cohort study (1/4) | CDASI: -34.6% (10/21)  Skin-VAS: -54.6% (9/21) | 5.5% (9/21) | IM (61.9%, 13/21) | 23.1 (13/13) | Infections (14/21); cardiovascular events (4/21); malignancy (3/21); skin eruptions (3/21); musculoskeletal system events (3/21); gastrointestinal events (3/21); febrile episodes (2/31); UTI (1/21); neuropathological events (1/21); worsening of pre-existing interstitial lung disease (1/21); worsening right knee contracture (1/21); focal lipoatrophy (1/31); skin infection (1/21); *E. coli* diarrhea (1/21) | 6.9 (13/13) |
|  |  |  |  | NIM (38.1%, 8/21) | 24 (8/8) |  | 7.5 (2/2) |
| Adalimumab (2.9%, 17/589) | Case report (2/3)  Retrospective study (1/3) | NR | NR | IM (100%, 17/17) | 18.4 (17/17) | NR | 12.5 (17/17) |
| Tocilizumab (2.3%, 14/589) | Case report (3/5)  Randomized controlled trial (1/5)  Case series (1/5) | NR | 6.6% (10/14) | IM (28.6%, 4/14) | 20 (3/4) | Infection (8/14); bilateral pulmonary emboli (1/14); cytomegalovirus infection (1/14); liver dysfunction (1/14) | 15.7 (3/4) |
|  |  |  |  | NIM (71.4%, 10/14) | 24 (10/10) |  | 6 (10/10) |
| Anakinra (0.5%, 3/589) | Case report (2/3)  Case series (1/3) | NR | NR | IM (100%, 3/3) | NR | Aspiration pneumonia (1/3) | 5 (1/3) |
| Anifrolumab (0.3%, 2/589) | Case report (2/2) | CDASI: -77.8% (1/2) | NR | IM (100%, 2/2) | 8.5 (2/2) | NR | 3.5 (2/2) |
| Dupilumab (0.3%, 2/589) | Case report (2/2) | NR | NR | IM (100%, 2/2) | 13 (2/2) | NR | 3.3 (2/2) |
| Ustekinumab (0.2%, 1/589) | Case report (1/1) | NR | NR | IM (100%, 1/1) | 72 (1/1) | NR | 18 (1/1) |
